# *Klebsiella pneumoniae* causes bacteremia using factors that mediate tissue-specific fitness and resistance to oxidative stress

**DOI:** 10.1371/journal.ppat.1011233

**Published:** 2023-07-18

**Authors:** Caitlyn L. Holmes, Alexis E. Wilcox, Valerie Forsyth, Sara N. Smith, Bridget S. Moricz, Lavinia V. Unverdorben, Sophia Mason, Weisheng Wu, Lili Zhao, Harry L. T. Mobley, Michael A. Bachman

**Affiliations:** 1 Department of Pathology, University of Michigan Medical School, Ann Arbor, Michigan, United States of America; 2 Department of Microbiology and Immunology, University of Michigan Medical School, Ann Arbor, Michigan, United States of America; 3 Bioinformatics Core Facility, School of Medicine, University of Michigan, Ann Arbor, Michigan, United States of America; 4 Research Institute, Beaumont Hospital, Royal Oak, Michigan, United States of America; Children’s Hospital Boston, UNITED STATES

## Abstract

Gram-negative bacteremia is a major cause of global morbidity involving three phases of pathogenesis: initial site infection, dissemination, and survival in the blood and filtering organs. *Klebsiella pneumoniae* is a leading cause of bacteremia and pneumonia is often the initial infection. In the lung, *K*. *pneumoniae* relies on many factors like capsular polysaccharide and branched chain amino acid biosynthesis for virulence and fitness. However, mechanisms directly enabling bloodstream fitness are unclear. Here, we performed transposon insertion sequencing (TnSeq) in a tail-vein injection model of bacteremia and identified 58 *K*. *pneumoniae* bloodstream fitness genes. These factors are diverse and represent a variety of cellular processes. *In vivo* validation revealed tissue-specific mechanisms by which distinct factors support bacteremia. ArnD, involved in Lipid A modification, was required across blood filtering organs and supported resistance to soluble splenic factors. The purine biosynthesis enzyme PurD supported liver fitness *in vivo* and was required for replication in serum. PdxA, a member of the endogenous vitamin B6 biosynthesis pathway, optimized replication in serum and lung fitness. The stringent response regulator SspA was required for splenic fitness yet was dispensable in the liver. In a bacteremic pneumonia model that incorporates initial site infection and dissemination, splenic fitness defects were enhanced. ArnD, PurD, DsbA, SspA, and PdxA increased fitness across bacteremia phases and each demonstrated unique fitness dynamics within compartments in this model. SspA and PdxA enhanced *K*. *pnuemoniae* resistance to oxidative stress. SspA, but not PdxA, specifically resists oxidative stress produced by NADPH oxidase Nox2 in the lung, spleen, and liver, as it was a fitness factor in wild-type but not Nox2-deficient (*Cybb*^-/-^) mice. These results identify site-specific fitness factors that act during the progression of Gram-negative bacteremia. Defining *K*. *pneumoniae* fitness strategies across bacteremia phases could illuminate therapeutic targets that prevent infection and sepsis.

## Author summary

Gram-negative bacteremia is a deadly family of infections that initiate sepsis, a leading cause of global morbidity and mortality. Only a small number of Gram-negative species contribute to the majority of clinical bacteremia. *Klebsiella pneumoniae* is the second leading cause of Gram-negative bacteremia, and the third leading cause of overall bloodstream infection. *K*. *pneumoniae* is highly linked to hospital-associated infection with increasing antimicrobial resistance, endangering the most vulnerable patients. It is critical to understand the pathogenesis of *K*. *pneumoniae* bacteremia to better develop targets for future therapies that can prevent these deadly infections. Here, we define over 50 *K*. *pneumoniae* genes that support bloodstream fitness. These factors are diverse, support tissue-specific fitness, and increase bacterial resistance to oxidative stress. Our study is the first to systematically define *K*. *pneumoniae* factors enhancing bacteremia in a mammalian system. These results illuminate host-pathogen interactions during *K*. *pneumoniae* bacteremia that may be extended to additional Gram-negative species.

## Introduction

Bacteremia, the presence of bacteria in the bloodstream, can initiate sepsis. Defined as immune dysregulation resulting in organ dysfunction, sepsis is a significant cause of global morbidity and mortality [1–3]. Gram-negative species underlie about half of clinical bacteremia cases and are emerging in dominance [4]. To establish bacteremia, Gram-negative species follow three phases of pathogenesis. First, bacteria invade or colonize tissues that serve as initial sites of infection. Second, bacteria cross host barriers unique to the initial site and disseminate into the blood. Third, bacteria must survive in the bloodstream by exercising metabolic flexibility and avoiding clearance in filtering organs like the spleen and liver [2]. Defining bacterial factors that enhance bacteremia is a step toward treating this family of deadly infections.

*Klebsiella pneumoniae* is the second leading cause of Gram-negative bacteremia [4]. Repeatedly classified as a pathogen of urgent concern by the World Health Organization [5,6], infection with *K*. *pneumoniae* is particularly problematic due to a high association with mortality and antimicrobial resistance [7,8]. *K*. *pneumoniae* is highly linked to hospital associated infection, particularly pneumonia [9]. Accordingly, *K*. *pneumoniae* lung fitness mechanisms have been extensively described and include a wide variety of factors such as capsular polysaccharide, branched chain amino acid synthesis, and production of citrate synthase [10–15]. *K*. *pneumoniae* mechanisms enhancing dissemination have been less thoroughly described but are likely partially dependent on ADP-heptose biosynthesis and siderophores [16,17]. The host hypoxia-inducible factor 1α (HIF-1α) in lung epithelial cells also promotes dissemination, although interactions at this step are not well understood [18].

*K*. *pneumoniae* factors involved in the last phase of bacteremia, survival in the blood and filtering organs, remain incompletely defined. *In vitro* studies using human serum identified *K*. *pneumoniae* capsule biosynthesis and lipopolysaccharide (LPS) O-antigen as essential for complement resistance [19,20]. Human serum studies also demonstrated that vitamin biosynthesis and protein translocation using the *tat* system support maximum growth [20]. Modeling bacteremia using *Galleria mellonella* further confirmed a role for capsular polysaccharide, LPS, cellular envelope integrity, and iron acquisition systems during infection [21]. While *in vitro* studies have consistently identified subsets of *K*. *pneumoniae* genes required for serum growth and complement evasion, these models cannot define factors that influence fitness in blood filtering organs. Since bloodstream fitness involves both replication and evasion of clearance, some factors may be dispensable for serum growth but perpetuate bacteremia through interactions within tissues. For example, GmhB, an ADP-heptose biosynthesis enzyme required to produce intact LPS inner core, is dispensable for serum growth and lung fitness but is required for fitness in the liver and spleen during bacteremia [16]. Thus, GmhB is an example of a fitness factor specific to the last phase of bacteremia and can only be observed *in vivo*. This demonstrates that approaches defining bloodstream survival mechanisms should incorporate systemic infection to fully reveal *K*. *pneumoniae* factors perpetuating bacteremia.

Additionally, *in vivo* models can uncover host-pathogen interactions during bacteremia. To eliminate bacteria, immune cells may induce an oxidative stress response. NADPH oxidases, specifically the phagocytic Nox2, generate bursts of reactive oxygen species (ROS) that are frontline host defense mechanisms during infection [22]. Despite the importance of this response, *K*. *pneumoniae* factors enhancing resistance to ROS-mediated stress have not been detected in blood filtering organs. Immune responses can also vary across organs as tissue-resident cells can have differential interactions with bacteria, including *K*. *pneumoniae* [23]. Varying replication rates of multiple Gram-negative species across tissues during bacteremia highlights that host-pathogen interactions are not uniform between sites [24]. Thus, defining bacterial site-specific fitness mechanisms can illuminate host defense strategies.

Our group has used transposon insertion-site sequencing (TnSeq) to define bacterial genes required for bloodstream fitness in Gram-negative species including *Escherichia coli*, *Serratia marcescens*, *Citrobacter freundii*, *Acinetobacter baumannii*, and *Proteus mirabilis* [25–30]. These studies defined an array of bacterial genes enhancing bloodstream fitness. Metabolic flexibility emerged as a prominent feature by which Gram-negative species survive in the blood. Interestingly, no factors have been identified that are universally required across all six species. This highlights that bacteremia may require a species-specific arsenal of fitness mechanisms. Considering the dominance of *K*. *pneumoniae* infections in clinical settings and resulting high mortality rates [7–9,31], it is critical to define mechanisms by which this specific pathogen perpetuates bacteremia. Additionally, *K*. *pneumoniae* mechanisms of pathogenesis apart from the lung are largely unknown and gaining knowledge of these processes will allow insight into this pathogen.

To define *K*. *pneumoniae* bacteremia factors influencing bloodstream survival, we performed TnSeq using a mammalian model of intravascular bacteremia and revealed 58 diverse genes that enhance splenic fitness. Validation studies demonstrate that bacteremia is enhanced by a set of factors relaying tissue-specific fitness in the spleen, serum, and liver. This study is the first to systematically identify *K*. *pneumoniae* strategies for *in vivo* bloodstream fitness.

## Results

### Transposon insertion-site sequencing during *K*. *pneumoniae* bacteremia reveals diverse mechanisms enhancing bloodstream fitness

**Fig 1:**
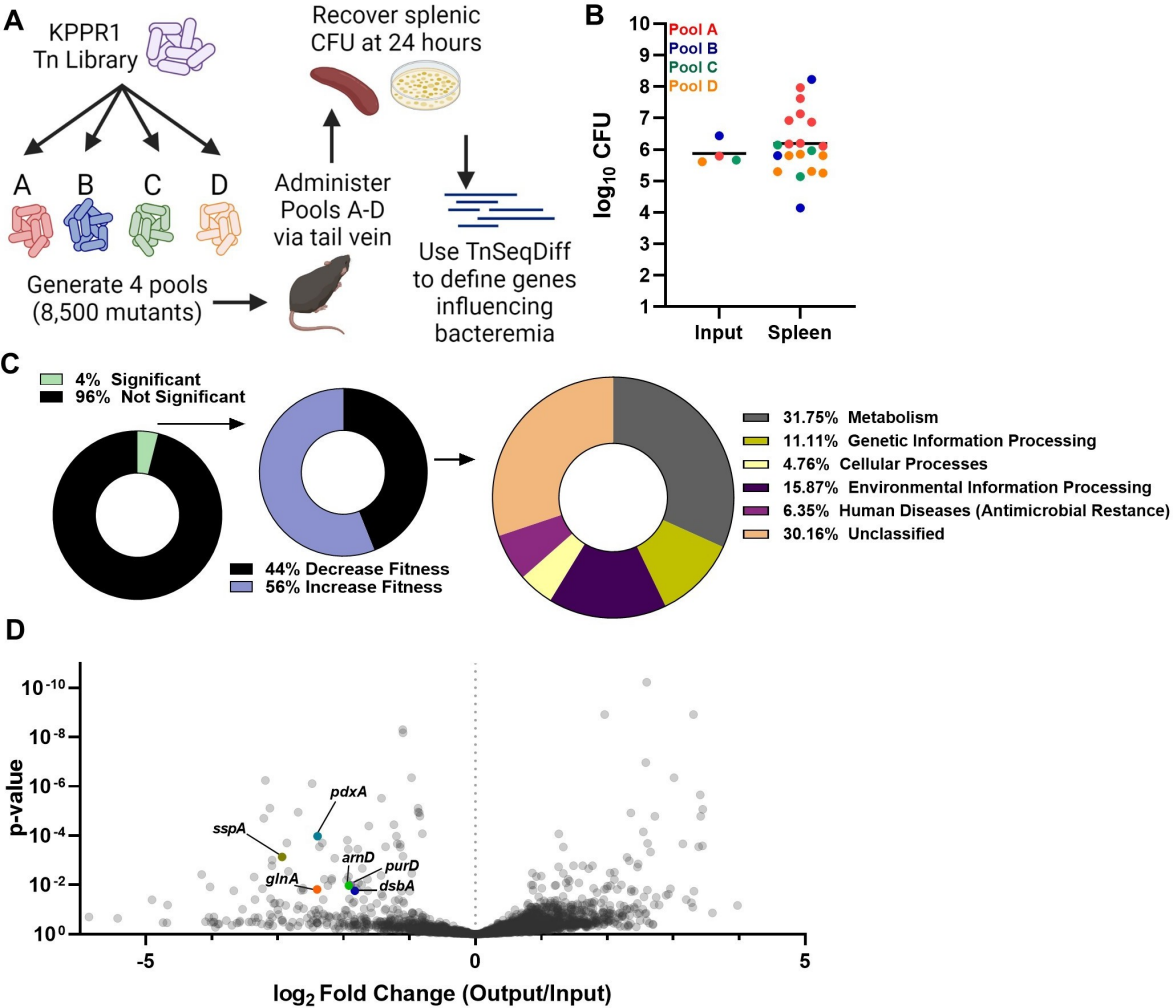
Transposon insertion-site sequencing (TnSeq) reveals that *K*. *pneumoniae* bacteremia is enhanced by a set of genes representing diverse fitness mechanisms. (A) Overview of *K*. *pneumoniae* bacteremia TnSeq. A KPPR1 transposon library was divided into four pools containing 8,500 unique insertions and administered to mice via the tail vein at a 1x10^6^ CFU dose. After 24 hours, splenic CFU was recovered, and DNA was sequenced. The TnSeqDiff pipeline determined genes influencing fitness. This illustration was created with BioRender.com. (B) The input and splenic CFU burden for each pool and mouse represented in TnSeq, with mean organ CFU colonization represented by the bar. (C) Genes represented in TnSeq (~3,800 genes) that were defined as influencing infection (132 genes), and primary KEGG orthology for genes increasing *K*. *pneumoniae* fitness during bacteremia (58 genes). (D) A volcano plot displaying genes represented in the TnSeq screen with log_2_ fold change (x-axis) and p-value (y-axis) defined by the TnSeqDiff pipeline. The genes selected for further study, *arnD*, *purD*, *dsbA*, *sspA*, and *pdxA*, are highlighted.

To define *K*. *pneumoniae* factors influencing bacteremia pathogenesis, transposon-insertion site sequencing (TnSeq) was performed using a previously described KPPR1 library containing ~25,000 unique mutations [11,32]. To model the third phase of bacteremia, survival in the blood and filtering organs, an intravascular model was used with inoculation of mice via tail vein injection (Fig 1A). This model bypasses the first two phases of bacteremia, initial site infection and dissemination, and allows for direct examination of bloodstream fitness [16,33]. Because *K*. *pneumoniae* abundance in the blood 24 hours after tail vein injection is incredibly low, and often undetectable, the spleen was selected to represent *K*. *pneumoniae* fitness in the third phase of bacteremia. To define potential experimental bottlenecks to the spleen in this model, a fitness-neutral mutant was competed against wild-type KPPR1 at ratios of 1:1, 1:5,000, and 1:10,000. At the 1:10,000 ratio, the mutant was recovered in significantly lower abundance than KPPR1 indicating potential stochastic loss at this ratio (S1 Fig). In contrast, loss was minimal at the 1:5,000 dilution. Therefore, 1:8,500 was selected as the target input complexity to represent approximately 1/3 of the total mutants. To increase the chance of sampling all mutants in the library, the KPPR1 transposon library was used to generate four pools, and each pool (Pools A-D) was administered to 10 mice.

Twenty-four hours post-inoculation, total splenic CFU was recovered, DNA was extracted and subjected to sequencing, and the abundance of unique transposon insertions was compared to the inoculum as described in the Materials and Method section. The actual number of unique transposon mutations varied by pool, ranging from ~9,350–12,450 insertions. Mean splenic colonization was roughly 1x10^6^ CFU (Fig 1B) and mouse mortality between pools varied widely from 0–70%. As a result, spleen samples from 8, 3, 3, and 6 mice within pools A, B, C, and D, respectively, were used for analysis. Within the input pools, ~3,800 of the estimated 4,312 non-essential KPPR1 genes contained transposon insertions (S1 Table). 132 genes, 4% of those within the study, were identified as significantly influencing *K*. *pneumoniae* fitness during bacteremia (Fig 1C). Of significant hits, 58 (44%) genes with transposon insertions resulted in lower recovery compared to the input, revealing bacterial factors enhancing bacteremia. KEGG annotation summaries for these genes demonstrated that a diverse set of *K*. *pneumoniae* factors enhance bloodstream survival (Table 1). The most highly represented genetic function was metabolism (Fig 1C). Defined by KEGG orthology, no single metabolism type was dominant and synthesis of carbohydrates, nucleotides, amino acids, vitamins, and other substrates supported bloodstream fitness (S2 Fig). In addition, 56% of genes with transposon insertions resulted in higher recovery compared to the input (S2 Table), a pattern which indicates a set of genes that suppress pathogenesis since mutations led to enrichment after infection. Since the goal of the present study was to define genes enhancing bacteremia, this group was not analyzed further. Most of these hits have unclassified functional KEGG annotations. Of the hits with annotated functions, metabolic pathways were highly represented (S2 Fig).

**Table 1:** *K*. *pneumoniae* splenic fitness factors.

Locus ID (VK-55_#)	Gene	log_2_ Fold Change (Spleen/Input Reads)	*p* value	GenBank Definition
VK055_2575	*arcA*	-4.908	0.040	*arcA* transcriptional dual regulator
VK055_2352	*gmhB (yaeD)*	-4.156	0.004	D,D-heptose 1,7-bisphosphate phosphatase
VK055_3845		-4.026	0.012	hypothetical protein
VK055_1303		-3.669	0.017	EAL domain protein
VK055_3527		-3.359	0.005	mannitol dehydrogenase Rossmann domain protein
VK055_4296	*recB*	-3.346	0.039	exodeoxyribonuclease V, beta subunit
VK055_3731	*crp*	-3.232	0.014	CRP transcriptional dual regulator
VK055_3327	*gidA*	-3.212	0.000	tRNA uridine 5-carboxymethylaminomethyl modification enzyme
VK055_3630	*arnF*	-3.188	0.000	undecaprenyl phosphate-alpha-L-ara4N flippase subunit ArnF
VK055_2866	*rplI*	-3.119	0.000	ribosomal protein L9
VK055_2766	*yadF2*	-3.108	0.036	tRNA-Leu
VK055_4748		-3.089	0.002	PTS enzyme I
VK055_4686	*purM*	-3.085	0.001	phosphoribosylformylglycinamidine cyclo-ligase
VK055_3849	*sspA*	-2.933	0.001	stringent starvation protein A
VK055_3865	*rpoN*	-2.864	0.000	RNA polymerase sigma-54 factor
VK055_3832	*argR*	-2.839	0.003	arginine repressor
VK055_3183		-2.693	0.000	polysaccharide biosynthesis family protein
VK055_3142	*tatC*	-2.646	0.016	twin arginine-targeting protein translocase TatC
VK055_3343	*pstC*	-2.620	0.013	phosphate ABC transporter, permease protein PstC
VK055_3357	*trmE*	-2.612	0.016	tRNA modification GTPase TrmE
VK055_2585	*ccmA7*	-2.477	0.000	heme ABC exporter, ATP-binding protein CcmA
VK055_3295	*glnA*	-2.405	0.015	glutamine synthetase, type I
VK055_2525	*pdxA2 (pdxA)*	-2.398	0.000	4-hydroxythreonine-4-phosphate dehydrogenase
VK055_0077	*msbB*	-2.368	0.000	lipid A biosynthesis (KDO)2-(lauroyl)-lipid IVA acyltransferase
VK055_3612		-2.326	0.000	phosphate transporter family protein
VK055_4701	*purC*	-2.311	0.039	phosphoribosylaminoimidazolesuccinocarboxamide synthase
VK055_3592	*pqqL*	-2.293	0.006	insulinase family protease
VK055_1868	*dacC*	-2.241	0.011	penicillin-binding protein 6
VK055_4583		-2.184	0.007	23S rRNA pseudouridine synthase
VK055_1359		-2.180	0.005	NADH dehydrogenase II
VK055_3293	*typA*	-2.136	0.001	GTP-binding protein TypA/BipA
VK055_3301	*polA*	-1.959	0.006	DNA polymerase I, 3’ — 5’ polymerase, 5’ — 3’ and 3’ — 5’ exonuclease
VK055_4601	*nadB*	-1.944	0.000	L-aspartate oxidase
VK055_1352	*mfd*	-1.932	0.000	transcription-repair coupling factor
VK055_3186	*rfbA*	-1.927	0.005	glucose-1-phosphate thymidylyltransferase
VK055_3626	*arnD*	-1.921	0.011	putative 4-deoxy-4-formamido-L-arabinose-phosphoundecaprenol deformylase ArnD
VK055_3086	*purH*	-1.914	0.037	phosphoribosylaminoimidazolecarboxamide formyltransferase/IMP cyclohydrolase
VK055_3088	*purD*	-1.897	0.010	phosphoribosylamine—glycine ligase
VK055_3046	*ubiC*	-1.840	0.048	chorismate lyase
VK055_3303	*dsbA*	-1.832	0.017	DSBA-like thioredoxin domain protein
VK055_4048	*exbB*	-1.812	0.014	tonB-system energizer ExbB
VK055_2155	*tig*	-1.787	0.000	trigger factor
VK055_3158	*recQ*	-1.775	0.008	ATP-dependent DNA helicase RecQ
VK055_2385	*glnD*	-1.729	0.010	protein-P-II uridylyltransferase
VK055_3137	*fre*	-1.718	0.002	NAD(P)H-flavin reductase
VK055_4099	*gshB*	-1.696	0.039	glutathione synthase
VK055_3257	*cpxR*	-1.641	0.006	response regulator
VK055_4938		-1.632	0.017	hypothetical protein
VK055_4619	*purL*	-1.619	0.000	phosphoribosylformylglycinamidine synthase
VK055_3184	*wecE*	-1.434	0.004	TDP-4-keto-6-deoxy-D-glucose transaminase familyprotein
VK055_3858	*arcB*	-1.426	0.023	aerobic respiration control sensor protein ArcB
VK055_5102	*iroC*	-1.409	0.029	lipid A export permease/ATP-binding protein MsbA
VK055_3823		-1.365	0.000	hypothetical protein
VK055_3817		-1.221	0.044	EAL domain protein
VK055_4161	*dsbC*	-1.167	0.039	thiol:disulfide interchange protein
VK055_2237		-1.120	0.018	ABC transporter transmembrane region 2 family protein
VK055_3679		-1.101	0.047	Glycerol-3-phosphate dehydrogenase
VK055_3152	*yedA*	-0.999	0.018	carboxylate/amino acid/amine transporter family protein

Six of the 58 genes enhancing bacteremia were then selected for characterization based on representing diverse cellular functions, potential conserved Enterobacterales bacteremia fitness factors [26,27], or being unique to *K*. *pneumoniae* (Fig 1D). Specifically, *arnD* was selected since it transcribes a member of a Lipid A modification system and other LPS modifications have been identified as enhancing bacteremia [16]. Multiple purine biosynthesis genes enhanced spleen fitness, and *purD* was selected as the hit furthest upstream within this pathway. DsbA, a member of a disulfide bond formation and secretion system, enhances uropathogenesis in *E*. *coli* and was selected for study within *K*. *pneumoniae* bacteremia [34]. SspA is a regulator of the stringent starvation response, linked to multiple pathways of cell stress regulation, and was selected for investigation into splenic stress. PdxA is a member of the endogenous vitamin B6 biosynthesis cascade and was selected since it has no known links to bacteremia pathogenesis. Lastly, GlnA is a glutamine synthetase enhancing splenic fitness in other species [25–27]. Transposon mutants for each gene of interest were selected from a KPPR1 ordered library [35] and growth was observed in rich medium (LB). Apart from *glnA*, *in vitro* replication was not influenced by mutations within these genes, meaning that contributions to bacteremia were likely unrelated to basic cellular replication in nutrient rich environments (S3 Fig). *K*. *pneumoniae* bacteremia hits also had varying effects on hypermucoviscosity (S3 Fig). Mutations within *arnD* significantly reduced, as previously reported [35], while mutations within *glnA* and *pdxA* enhanced, hypermucoviscosity.

### *K*. *pneumoniae* bacteremia pathogenesis is perpetuated by factors that relay site-specific fitness

To validate the bacteremia TnSeq results, the five genes of interest that did not demonstrate *in vitro* replication defects were further analyzed: *arnD*, *purD*, *dsbA*, *sspA*, and *pdxA*. Each gene was represented by 3–5 unique transposon insertions within the TnSeq study, and insertion level analysis for each gene’s unique mutations across individual animals demonstrated consistent loss in splenic abundance compared to the input (S4 Fig).

**Fig 2:**
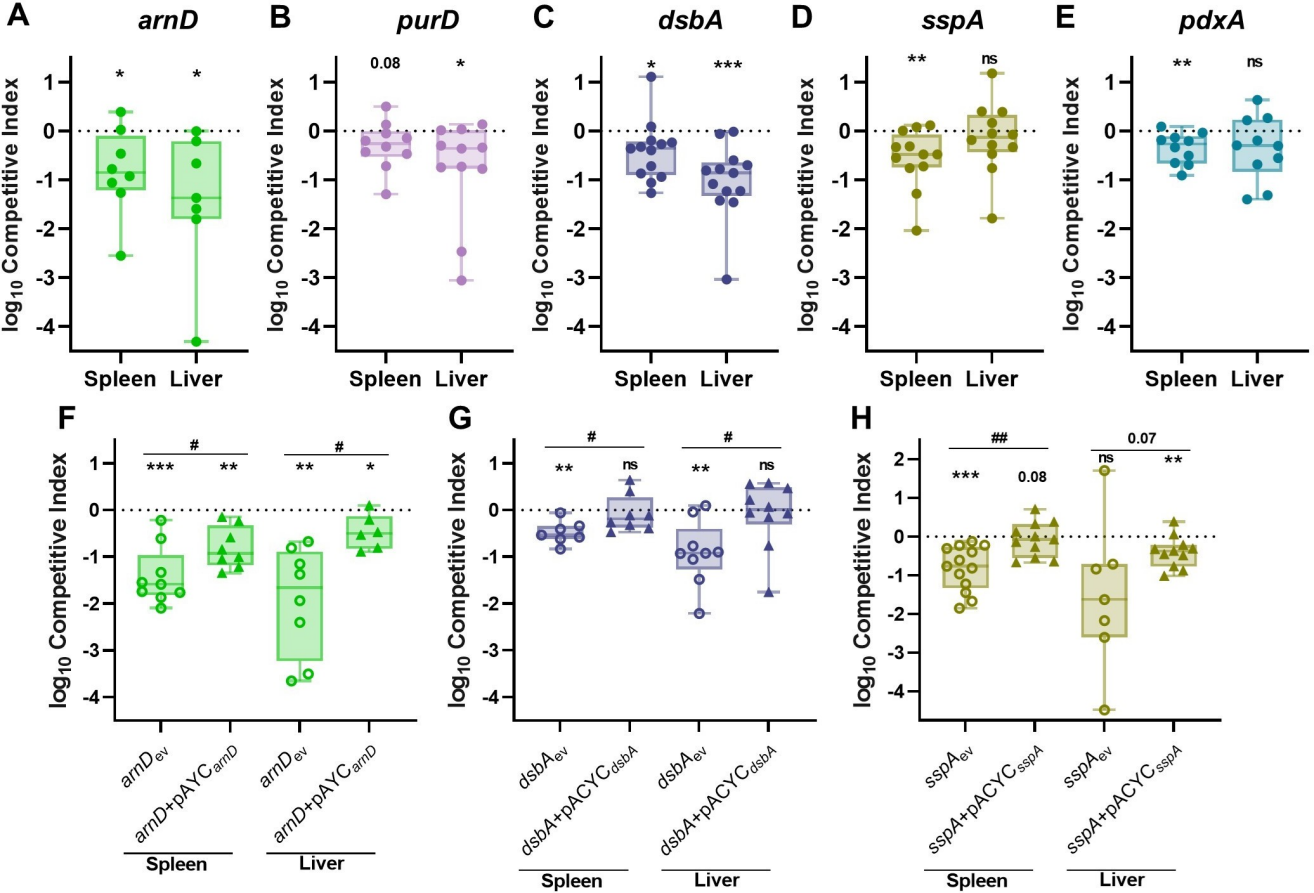
*K*. *pneumoniae* bacteremia fitness factors directly relay tissue-specific fitness advantages. Five factors indicated by TnSeq as significantly enhancing bacteremia were selected for *in vivo* validation using the tail vein injection model. The 1:1 inoculum consisted of KPPR1 and transposon mutants for (A) *arnD*, (B) *purD*, (C) *dsbA*, (D) *sspA*, or (E) *pdxA*. Competitions were also performed using strains carrying the empty pACYC vector (_ev_) or complementation provided on pACYC184 under native promoter control for (F) *arnD* (*arnD*+pACYC_*arnD*_), (G) *dsbA* (*dsbA*+pACYC_*dsbA*_), or (H) *sspA* (*sspA*+pACYC_*sspA*_). The log_10_ competitive index at 24 hours post infection is displayed for individual mice with bars representing the median and interquartile range. For all, *p<0.05, **p<0.01, ***p<0.001 by one sample *t* test with a hypothetical value of zero; for (F-H) ^#^p<0.05, ^##^p<0.01 by unpaired *t* test. For each group, n*≥*8 mice in at least two independent trials.

For *in vivo* validation of the TnSeq study, individual transposon mutants were competed against KPPR1 at a 1:1 ratio in the intravascular bacteremia model. ArnD had the greatest influence on bacteremia, demonstrating an *in vivo* fitness requirement in both the spleen and liver (Figs 2A and S5). A mutant in *purD* approached but did not reach significance (*P* = 0.08) for splenic fitness defects but was significantly defective in the liver (Figs 2B and S5). DsbA was required in both compartments but had a larger influence on liver fitness (Figs 2C and S5). In contrast, SspA was required in the spleen but was dispensable in the liver (Figs 2D and S5). PdxA had a modest but significant fitness defect in the spleen (Figs 2E and S5). To validate our statistical approach, we competed KPPR1 against a kanamycin marked KPPR1 strain (KPPR1_Kan_) and compared competitive indices between this fitness neutral competition and genes of interest from the study (S6 Fig). This alternative method supported our original findings. Notably, each bacteremia factor enhanced fitness in a distinct manner across blood filtering organs. Factors displayed unique patterns of tissue-specific fitness with some, like ArnD and DsbA being required across organs, while PurD, SspA, and PdxA were only required in one organ.

To confirm that fitness defects were specifically related to disruption of the genes of interest, *arnD*, *dsbA*, and *sspA* were complemented *in trans* as mutations in these genes resulted in large spleen and liver fitness defects. Complementation of *arnD* significantly alleviated bacteremia fitness defects in comparison to *arnD*_ev_, demonstrating that *arnD* enhances splenic and liver fitness (Figs 2F and S5). However, the *arnD+*pACYC_*arnD*_ strain continued to demonstrate lower fitness in relation to KPPR1_ev_. This is likely due to polar effects on downstream genes of the collective function of the *arn* system, consisting of other Lipid A modifying enzymes [36]. Indeed, another member of the *arn* operon, *arnF*, was a TnSeq hit, further validating a role for this system in bacteremia (Table 1). DsbA directly enhances spleen and liver fitness as restoration of *dsbA* in the mutant strain significantly alleviates defects and restores fitness that is comparable to the wild-type strain (Figs 2G and S5). SspA is a regulator of the complex stringent starvation response and *sspA* mutations lead to higher susceptibility to many environmental stressors [37]. SspA complementation (*sspA+*pACYC_*sspA*_) significantly ameliorated *K*. *pneumoniae* splenic fitness defects (Figs 2H and S5). Therefore, *in vivo* complementation of the three selected factors restored *K*. *pneumoniae* bacteremia fitness and specifically highlight genes that influence spleen and liver fitness. This confirms that the TnSeq study revealed multiple factors that directly enhance bacteremia as site-specific factors.

### *K*. *pneumoniae* utilizes multiple strategies to enhance site-specific fitness during bacteremia, including metabolic flexibility, serum resistance, and LPS modification

**Fig 3:**
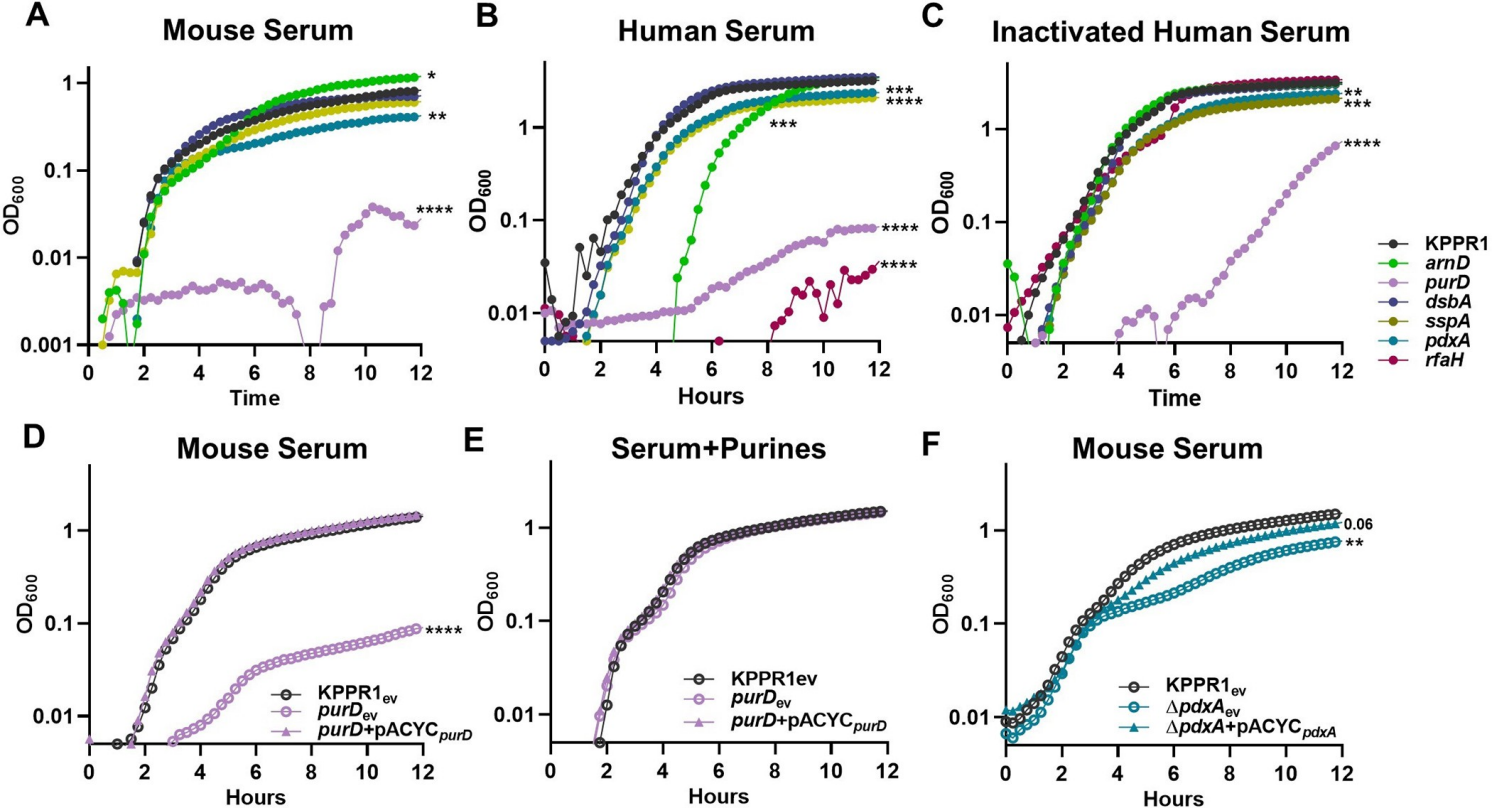
*K*. *pneumoniae* serum replication requires purine synthesis and is maximized by endogenous vitamin B6 biosynthesis. *K*. *pneumoniae* strains were grown in M9 salts supplemented with (A) 10% mouse serum or 20% human serum that was either (B) active or (C) heat inactivated. *K*. *pneumoniae* strains carrying the empty vector pACYC (_ev_) or pACYC expressing (D,E) *purD* (*purD*+pACYC_*purD*_) or (F) *pdxA* (Δ*pdxA*+pACYC_*pdxA*_) were grown in 10% mouse serum (D, F). Chemical complementation for *purD* was measured by supplementation of 1mM purines prior to growth (E). For all, the OD_600_ was measured every 15 minutes for 12 hours. Differences in growth compared to KPPR1 or KPPR1_ev_ were detected by area under the curve using a one-way ANOVA with Dunnett’s multiple comparison for each strain compared to wild-type; *p<0.05, **p<0.01, ***p<0.001, ****p<0.0001. For each group, n = 3 in independent trials.

Since *in vivo* validation revealed distinct fitness factor contributions to tissue-specific fitness, individual bloodstream compartments were investigated to determine relevant interactions during infection. Bacterial replication occurs across compartments during bacteremia, and metabolic flexibility is critical to growth in the bloodstream [24–26]. To test contributions to growth in serum, each of the five validated bacteremia fitness factors were assessed for *in vitro* replication in mouse serum (Figs 3A and S7). Purine biosynthesis, mediated by PurD, was required for growth in murine and human serum as previously described [21,38]. This defect was not explained by complement resistance, as the *purD* mutant had a similar growth defect in both active and heat inactivated serum (Figs 3B–3C and S7). Genetic complementation of *purD* restored the ability for serum growth compared to the wild-type or *purD* strains carrying an empty complementation vector (Figs 3D and S7). Purines were sufficient to restore serum growth as exogenous purine supplementation ameliorated *purD* defects (Figs 3E and S7). Vitamin B6 biosynthesis also maximized replication in murine and human serum, as a *pdxA* mutant showed a mild defect (Fig 3A–3C). Genetic complementation of *pdxA* restored normal replication compared to wild-type and *pdxA* strains carrying the empty complementation vector (Figs 3F and S7). This indicates that the serum is a nutrient restricted environment and *K*. *pneumoniae* must endogenously produce key metabolites including purines and vitamin B6 to enable bloodstream replication.

**Fig 4:**
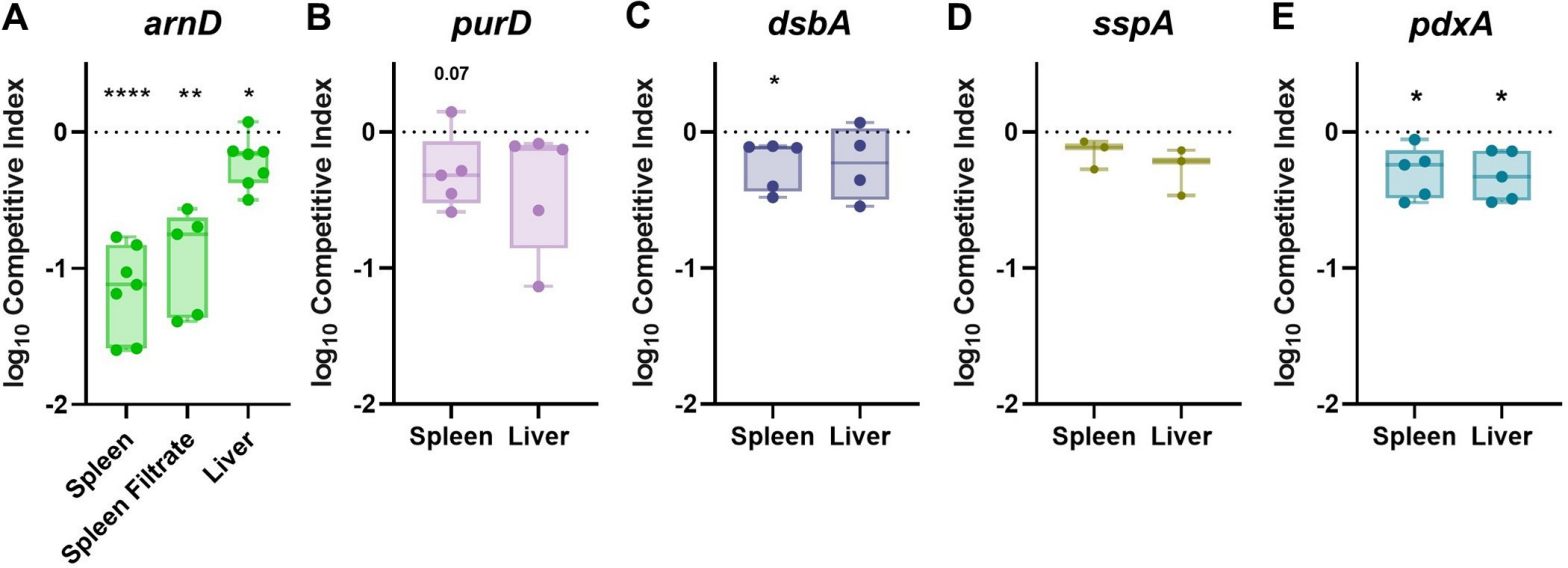
*K*. *pneumoniae* bacteremia factors convey fitness advantages through tissue-specific interactions within blood-filtering organs. *Ex vivo* competitions were performed using uninfected murine spleen or liver homogenate with a 1:1 mixture of KPPR1 and transposon mutants for (A) *arnD*, (B) *purD*, (C) *dsbA*, (D) *sspA*, (E) or *pdxA*. The log_10_ competitive index compared to wild-type KPPR1 at 3 hours post incubation is displayed for individual mice with bars representing the median and interquartile range. *p<0.05, **p<0.01, ****p<0.0001 by one-sample *t* test with a hypothetical value of zero and n = 3–7 with points representing mice in independent experiments.

ArnD is a member of a well-described LPS modification system (*arn* operon) that covalently attaches arabinose residues onto Lipid A [36]. Mutations in *arnD* render *K*. *pneumoniae* significantly less hypermucoviscous and reduce capsular polysaccharide production (S3 Fig, [35]). In active human serum, the *arnD* mutant was defective for growth, which was attributable to complement-mediated killing as heat inactivation of human serum restored normal *arnD* replication (Figs 3B–3C and S7). A similar pattern was observed for the control strain *rfaH*, which lacks capsular polysaccharide and is more susceptible to killing by active human serum [11,16]. Interestingly, murine serum replication was enhanced in the absence of ArnD (Figs 3A and S7). Since ArnD was dispensable for murine serum replication yet required for *in vivo* fitness in blood filtering organs, its fitness contribution in the spleen and liver was investigated (Fig 4). Using spleen and liver organ homogenates from uninfected mice, *ex vivo* competitions were performed for KPPR1 and *arnD*. Both experienced growth in organ homogenates (S8 Fig). The *arnD* mutant had a subtle yet significant fitness defect in liver homogenate and a dramatic fitness defect in splenic homogenate (Figs 4A and S6). To define relevant splenic compartments, splenocytes were removed from the homogenate, leaving only the soluble fraction. Strikingly, the mutant was also defective in splenic filtrate. This indicates that ArnD increases protection against a soluble factor specifically found in the spleen.

### Splenic fitness is influenced by route of infection

While ArnD and PurD dramatically influenced spleen and serum fitness, respectively, the contributions of DsbA, SspA, and PdxA to splenic fitness were more subtle (Fig 2). DsbA and PdxA were linked to enhancing splenic fitness as *K*. *pneumoniae* fitness defects in *dsbA* and *pdxA* were reproducible in uninfected splenic homogenate, mirroring the finding during infection (Fig 4C, E). Notably, these results are subtle as comparisons to competitive indices with a fitness neutral strain did not reveal significant defects (S6 Fig). PurD and SspA fitness defects in the spleen were not observed in *ex vivo* assays (Fig 4B, D). This could be due to direct, intravascular bacteremia only representing the third phase of pathogenesis and not encompassing the entire infection progression.

**Fig 5:**
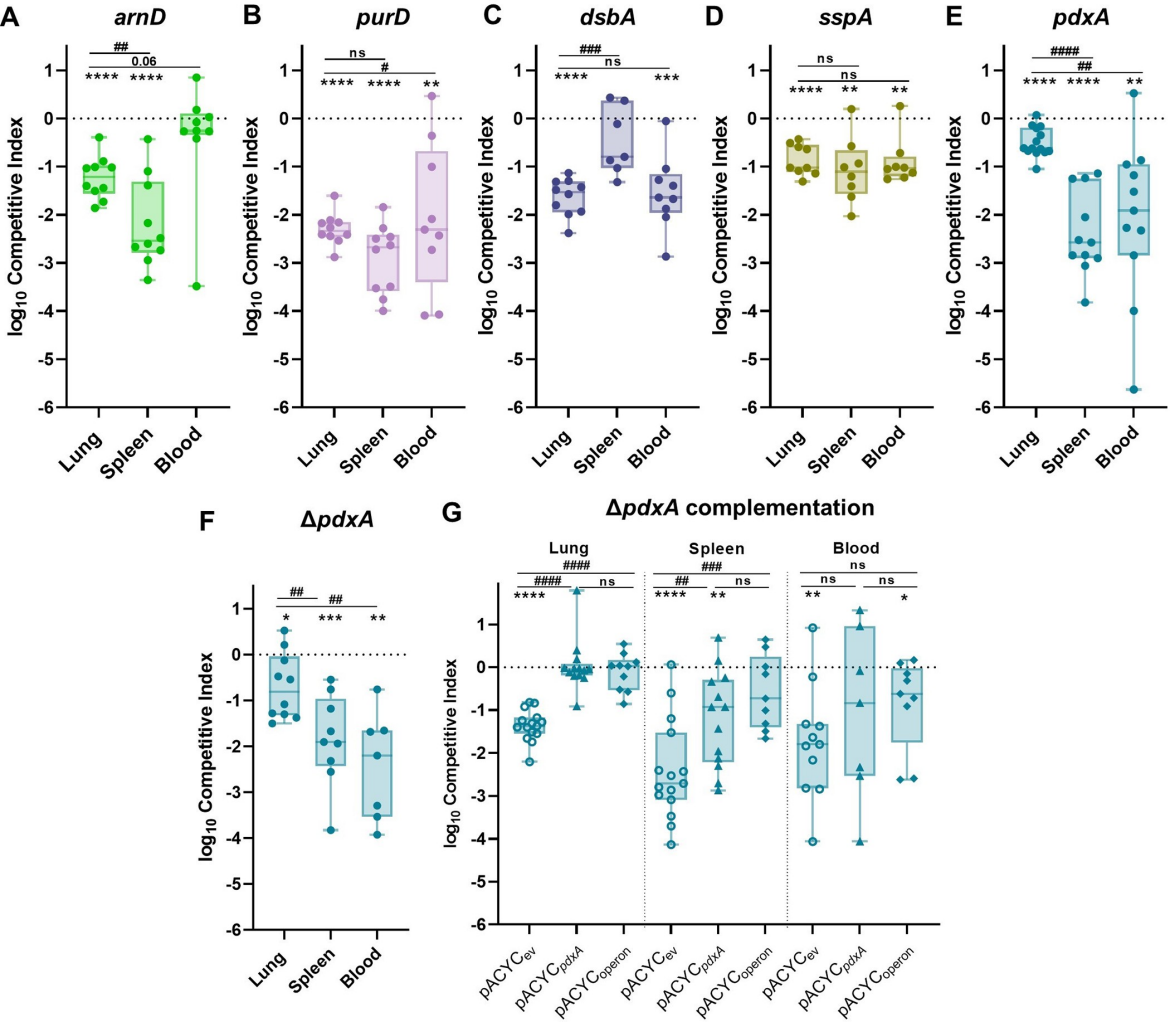
Primary site initiation of bacteremia enhances resolution of splenic fitness defects and illuminates requirements of factors across phases of pathogenesis. To model bacteremic pneumonia, mice were infected with 1x10^6^ CFU *K*. *pneumoniae*. Competitive infections were performed with a 1:1 mixture of KPPR1 and a transposon mutant for (A) *arnD*, (B) *purD*, (C) *dsbA*, (D) *sspA*, or (E) *pdxA* or (F) a *pdxA* knockout (Δ*pdxA*). (G) Competitions were also performed with strains carrying the empty pACYC vector (_ev_) or complementation provided on pACYC for *pdxA* only (pACYC_*pdxA*_) or *pdxA* and downstream members of the operon (pACYC_operon_). The log_10_ competitive index at 24 hours post infection is displayed for individual mice with bars representing the median and interquartile range. For all, *p<0.05, **p<0.01, ***p<0.001, ****p<0.0001 by one-sample *t* test with a hypothetical value of zero; ^#^p<0.05, ^##^p<0.01, ^###^p<0.001, ^####^p<0.0001 by unpaired *t* test. For each group, n≥10 mice in at least two independent infections.

To determine if ArnD, PurD, DsbA, SspA, and PdxA contributions to splenic fitness could be further resolved, *in vivo* competitions were repeated using a bacteremic pneumonia model which encompasses all three phases of bacteremia pathogenesis. As in the intravascular model, each factor promoted bacteremic pneumonia in a distinct manner. ArnD enhanced lung and spleen fitness but was dispensable in the blood (Fig 5A and S9). This finding supports Fig 3A, in which the *arnD* mutant had no growth defect in mouse serum, and Fig 4A, in which ArnD was required for splenic fitness. ArnD is likely most relevant to enhancing *K*. *pneumoniae* fitness during bacteremia in the context host threats inherent to tissues. PurD substantially supported lung fitness (Figs 5B and S9). Since *purD* mutants were so highly outcompeted in the lung, it is possible that spleen and blood fitness defects are an artifact of low bloodstream seeding by *purD* during these infections. Indeed, no *purD* mutants were recovered from the blood in this model which may indicate low bloodstream seeding or continued purine restriction during infection. DsbA also substantially supported lung fitness (Figs 5C and S9), linking DsbA to initial site fitness during bacteremia. However, there was no significant defect in the spleen in this model, in contrast with the tail vein model. SspA promoted fitness across sites at similar magnitudes (Figs 5D and S9), yet splenic fitness defects were more pronounced when incorporating a model with initial site infection and dissemination. Comparisons of competitive indices from bacteremia fitness factors to those from a fitness neutral competition further supported our findings. However, stringent bottlenecks during dissemination from the lung may confound perceived fitness defects in the blood as a fitness neutral strain had lower recovery in this model (S6 Fig). Thus, utilization of the bacteremic pneumonia model allows for greater resolution of splenic fitness defects and highlights that some factors, like PurD, and DsbA, may be most relevant to early phases of bacteremia.

PdxA significantly enhanced initial site fitness in the lung but had a striking effect on spleen and blood fitness during bacteremic pneumonia (Fig 5E). This pattern indicates potential contributions across all three phases of bacteremia as dissemination defects may worsen fitness at secondary sites when compared to initial sites [16]. Since the splenic fitness defect was most apparent in the bacteremic pneumonia model, *pdxA* complementation was performed in this infection. However, *in trans* complementation with *pdxA* and its upstream region did not restore fitness at any site (S9 Fig). *pdxA* is a member of a large, multifunctional operon and transposon insertions within *pdxA* may have polar mutations on downstream genes. To investigate the role of this operon in bacteremia, lambda red mutagenesis was used to generate a marked Δ*pdxA* strain which demonstrated similar fitness defects as the transposon *pdxA* mutant (Figs 5F and S9). Next, the Δ*pdxA* strain was complemented with *pdxA* alone (Δ*pdxA*+pACYC_*pdxA*_) or *pdxA* plus the downstream operon genes *ksgA*, *apaG* and *apaH* (Δ*pdxA*+pACYC_operon_). Complementation of *pdxA* alone was sufficient to restore lung fitness completely, and significantly improved fitness in the spleen but not the blood (Figs 5G and S9). Complementation of *pdxA* and downstream genes also improved fitness across sites (Figs 5G and S9), but did not significantly alleviate defects in comparison to complementation with *pdxA* only. This indicates that complementation of *pdxA* partially restores fitness defects even when downstream genes are included. Therefore, PdxA is a fitness factor in the lung and spleen.

### Splenic fitness is enhanced by oxidative stress resistance mechanisms

**Fig 6:**
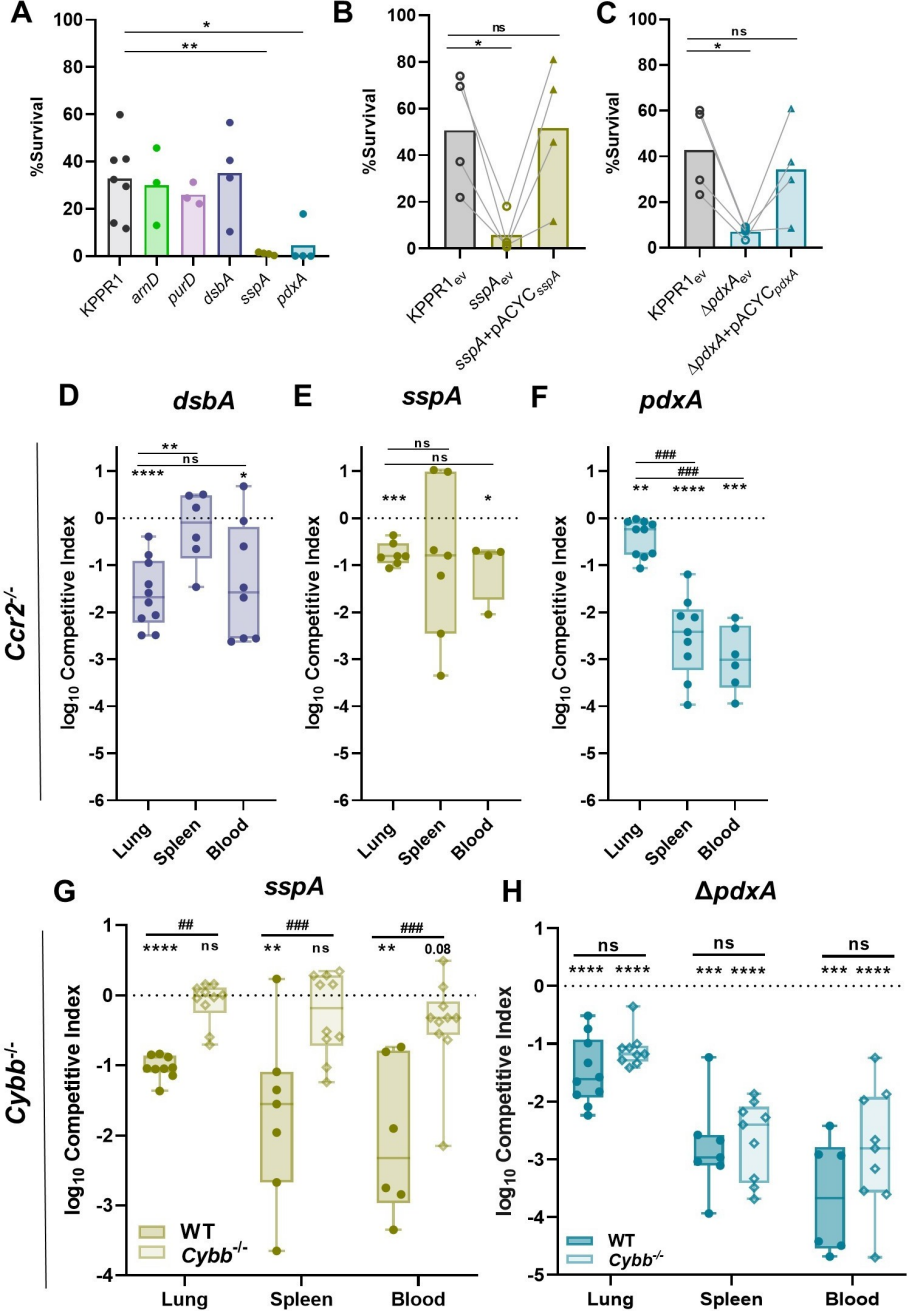
*K*. *pneumoniae* splenic fitness is maximized by factors relaying resistance to oxidative stress. (A) Resistance to oxidative stress was measured by incubating *K*. *pneumoniae* strains with H_2_O_2_. Complementation was performed by comparing strains carrying empty pACYC (_ev_) to those with pACYC expression of (B) *sspA* (*sspA*+pACYC_*sspA*_) or (C) *pdxA* (*pdxA*+pACYC_*pdxA*_). (D-H) In a model of bacteremic pneumonia, mice were infected with 1x10^6^ CFU *K*. *pneumoniae* containing a 1:1 mix of KPPR1 and a transposon mutant for (D) *dsbA*, (E) *sspA*, or (F) *pdxA* in *Ccr*2^-*/-*^ mice and (G) *sspA* or (H) Δ*pdxA* in *Cybb*^-*/-*^ mice. For (D-H), the log_10_ competitive index at 24 hours post infection is displayed for individual mice with bars representing the median and interquartile range. In (A-C), *p<0.05, **p<0.01 by one-way ANOVA with Dunnett’s correction for each strain compared to wild-type, n = 4 in independent trials. Bars represent the mean percent survival for each group. In B-C, lines connect samples from the same replicate. In (D-H), *p<0.05, **p<0.01, ***p<0.001, ****p<0.0001 by one-sample *t* test with a hypothetical value of zero and ^##^p<0.01, ^###^p<0.001 by unpaired *t* test. For each group, n≥7 mice in two independent infections.

Genes enhancing oxidative stress were widely represented in the significant TnSeq hits. For example, previous studies have demonstrated that mutations in *mtlD*, *arcA*, *recQ*, and *cpxR* increase susceptibility to hydrogen peroxide killing across multiple species [39–42]. Accordingly, survival of the *K*. *pneumoniae* mutants of interest after exposure to hydrogen peroxide was measured (Figs 6A and S10). The *sspA* and *pdxA* mutants had significant killing compared to KPPR1. SspA and PdxA were directly linked to oxidative stress as complementation of each restored the ability of *K*. *pneumoniae* to resist oxidative stress (Figs 6B–6C and S10).

Inflammatory CCR2^+^ monocytes are recruited to the lung during *K*. *pneumoniae* infection and are associated with clearance of bacteria and higher rates of murine survival [16,43,44]. To define if SspA and PdxA enhanced bacteremic pneumonia by interactions with CCR2^+^ monocytes and subsequent oxidative stress, infections were repeated in *Ccr2*^*-/-*^ mice [45]. Fitness defects across sites for each strain mirrored that of the wild-type mice and no differences in bacteremia fitness defects were observed between mouse genotypes (Figs 6D–6F and S10). Thus, DsbA, SspA, and PdxA mechanisms enhancing bacteremia fitness likely do not involve interactions with inflammatory monocytes at this early timepoint.

Nox2, phagocyte NADPH oxidase, generates a powerful ROS burst that serves as a main host defense against pathogens. To define if mechanisms mediating oxidative stress resistance were relevant *in vivo*, Nox2 knockout (*Cybb*^*-/-*^) mice were utilized [46]. Bacteremic pneumonia was repeated using competitive infections of KPPR1:*sspA* and KPPR1:*ΔpdxA* since these mutants showed remarkable susceptibility to oxidative stress and a fitness defect across compartments using this model. Unlike infections with wild-type mice, SspA was dispensable for fitness across compartments in *Cybb*^*-/-*^ mice (Figs 6G and S10). In contrast to SspA, PdxA was required for fitness across compartments in both wild-type and *Cybb*^*-/-*^ mice (Figs 6H and S10). Thus, SspA directly increases *K*. *pneumoniae* bacteremia fitness by promoting resistance to Nox2-mediated oxidative stress in the lung and spleen. This interaction is specific to SspA protection against Nox2-mediated oxidative stress as PdxA fitness defects are similar in both mouse backgrounds. Additionally, KPPR1 abundance is significantly increased in the lung of *Cybb*^*-/-*^ mice compared to wild-type mice, demonstrating that Nox2-mediated oxidative stress can partially control abundance of *K*. *pneumoniae* in the lung (S10 Fig). These data directly link together a *K*. *pneumoniae* bacteremia fitness factor to a host mechanism of clearance.

## Discussion

In this study, we combined murine intravascular bacteremia and TnSeq technology to define bacterial factors required for *K*. *pneumoniae* fitness during bloodstream infection. We found that *K*. *pneumoniae* bacteremia is enhanced by diverse factors, indicating that multiple mechanisms of pathogenesis are deployed to promote the phase of bloodstream survival. Bacteremia fitness factors are distinct in their ability to mediate site-specific fitness, with some genes being required for fitness in one site yet dispensable in others. Replication in serum was supported by purine biosynthesis and endogenous vitamin B6 biosynthesis, emphasizing nutrient restriction in this compartment. The ability of *K*. *pneumoniae* to resist Nox2-mediated oxidative stress during bacteremia was also critical, and we demonstrate direct and specific interactions between a bacteremia fitness factor, SspA, and ROS *in vivo*.

Comparing factors required for lung and bloodstream fitness demonstrates that many mechanisms are required across phases of bacteremia, yet others are phase specific. For *K*. *pneumoniae*, TnSeq has defined a broad spectrum of factors that enhance initial site fitness in the lung [11]. *K*. *pneumoniae* metabolic flexibility is required in the lung through biosynthesis of branched chain (*ilvC/D*) and aromatic (*aroE*) amino acids. However, these factors were not predicted by TnSeq as bloodstream fitness factors in the present study. Other pathways of metabolic flexibility were shared between studies. Multiple members of the purine biosynthesis pathway were predicted to enhance lung and bloodstream fitness, highlighting the importance of this pathway in more than one phase of bacteremia. In contrast, certain factors are unique to later phases of bacteremia. For example, the enzyme GmhB is dispensable for initial site fitness in the lung but was defined by this study and previous work to enhance bloodstream survival [16]. Therefore, therapies targeting specific factors may only be relevant in certain phases of disease. Notably, fitness defects at primary sites can confound perceived fitness defects at secondary sites. It is possible that host defenses in primary sites prime bacteria to survive at secondary sites, which may be the case for the *dsbA* mutant (Fig 5C) or elicit responses that are detrimental after dissemination. Models of bacteremia should be used in combination to probe fitness defects at each phase of pathogenesis to resolve these differences. By integrating models of direct bacteremia and primary site infection, we were able to study bacteremia fitness factors across distinct phases of pathogenesis.

Infection at primary sites further illuminated contributions of individual factors across phases of bacteremia. For example, DsbA substantially influenced lung fitness while contributing only subtly to splenic fitness across models. SspA and PdxA were also defined as lung fitness factors, and primary site fitness defects increased resolution of splenic defects. It is possible that site-specific stressors in the lung stimulate bacterial defenses that subsequently change splenic fitness. For example, host fatty acid oxidation elicited by other strains of *K*. *pneumoniae* in the lung results in bacterial adaptation to a new host microenvironment [13]. Perhaps these alterations significantly impact fitness at secondary sites, but this has not been experimentally validated for bacteremia. It is also possible that SspA and PdxA contribute to unknown mechanisms of lung dissemination, a *K*. *pneumoniae* process that is poorly understood. During pneumonia, *K*. *pneumoniae* siderophores elicit robust lung inflammation and stabilization of alveolar HIF-1α that results in dissemination to secondary sites [18]. Additional bacterial factors promoting dissemination through HIF-1α stabilization or through independent routes, and whether dissemination occurs actively or passively, are not well described and warrant further exploration.

Even within the phase of bloodstream survival, different fitness factors are important in different sites of infection. Despite active replication in both organs, *K*. *pneumoniae* abundance increases in the liver and decreases in the spleen during bacteremia [23,24]. Tissue-resident cells determine differential host responses across sites and can even influence the rate of bacterial clearance. In blood filtering organs, interactions with innate and adaptive immunity may regulate distinct fast and slow clearance mechanisms [47]. However, *K*. *pneumoniae* site-specific fitness has been minimally investigated. Our results demonstrate that multiple *K*. *pneumoniae* fitness factors contribute to bacteremia through tissue-specific mechanisms as all five factors selected for investigation demonstrated differential fitness patterns across organs (Fig 2). Some factors, like ArnD and DsbA, were required in more than one tissue, while PurD, SspA, and PdxA were required in only one. Thus, the results of this study both illuminate *K*. *pneumoniae* tissue-specific fitness strategies and define bacterial mutants that can be used as tools to explore host responses at distinct sites.

Gram-negative species actively replicate in the serum, and the biosynthesis of cellular building blocks is critical for survival in the blood since available nutrients differ by site [10,26]. Purine biosynthesis is required for replication in the blood [38], and our TnSeq results demonstrate that many members of the purine biosynthesis operon enhance *K*. *pneumoniae* bacteremia. Liver fitness was also enhanced by purine biosynthesis, yet splenic fitness did not reach statistical significance for this test. This tissue-specific dynamic for PurD indicates that purine availability may be more restricted in the serum and liver than the spleen. To our knowledge, the source of splenic purines remains unknown and whether the host actively restricts purine availability in the lung, serum, and liver is undefined.

Mechanisms of bacteremia fitness extended beyond metabolic flexibility as TnSeq revealed that many genes supporting splenic fitness were also associated with resistance to oxidative stress. Specifically, we discovered that SspA is required for oxidative stress resistance *in vivo*. Mice lacking the NADPH oxidase Nox2 (*Cybb*^*-/-*^) developed bacteremic pneumonia, yet SspA was dispensable for colonization across sites (Fig 6G) in this background. In contrast, SspA was required for fitness in wild-type mice, which produce a normal ROS response (Fig 5D). Importantly, protection against Nox2-mediated stress did not extend to all bacteremia fitness factors as PdxA, which mediated resistance to oxidative stress *in vitro*, retained a fitness defect in both wild-type and *Cybb*^*-/-*^ mice (Fig 6H). This “genetics-squared” approach, comparing infection phenotypes for both *K*. *pneumoniae* and host mutants allowed resolution of a novel and specific *in vivo* interaction. Thus, the stringent response regulator SspA enhances Gram-negative bacteremia by supporting resistance to oxidative stress, and ROS are responsible for eliciting some portion of *K*. *pneumoniae* control during bloodstream infection. However, relevant sources of ROS contributing to this response remain unclear. Monocyte-derived macrophages are likely a minimal source of ROS since mice lacking CCR2 do not demonstrate the alleviation of fitness defects in the absence of SspA observed in mice lacking Nox2. Future studies should determine the relevance of neutrophils and other immune cells in this interaction. Notably, SspA displayed tissue-specific fitness during intravascular bacteremia and was required in the spleen yet dispensable in the liver. These differences were not recapitulated *ex vivo* as *sspA* demonstrated no fitness defect in homogenate from either organ. Thus, the oxidative stress either arises from spleen-intrinsic factors that act over a longer time course or from spleen extrinsic factors or cells. In *Francisella tularensis* and *E*. *coli*, SspA regulates a large network of genes through contact with σ^70^ that alters RNA polymerase transcription of associated genes [48]. This dysregulation of a housekeeping sigma factor could create cellular disorder resulting in susceptibility to oxidative and environmental stressors [37]. Since the stringent starvation response is a complex system initiated to resist immune cells, redirect metabolism, and promote virulence [49], there could also be multiple additional mechanisms by which SspA supports tissue-specific fitness in addition to ROS resistance.

The use of *ex vivo* organ homogenate did allow resolution of ArnD-mediated tissue-specific fitness. The *arn* operon is well-documented in its role in modifying LPS Lipid A for the repulsion of cationic antimicrobial peptides [36,50]. While ArnD conveyed a significant fitness advantage in the liver and spleen, a surprising finding was that ArnD also conveyed a fitness advantage in the soluble splenic fraction. The fitness defect in the absence of ArnD is likely due to the secretion of an unidentified factor by splenocytes. Together, use of *ex vivo* organ homogenate suggests that factors inherent to individual tissues may inhibit *K*. *pneumoniae* growth.

A limitation of this study is the investigation of only a small subset of genes revealed as enhancing infection. Remarkably, genes defined by TnSeq as influencing bacteremia both supported and suppressed infection. This study focused on factors encoded by genes in which mutations decreased bacteremia fitness. However, other genes in which mutations led to greater *K*. *pneumoniae* bacteremia fitness may represent factors that are unfavorable for the bacteria during infection. This subset is also highly diverse and should be mined for future studies further understanding bacteremia fitness dynamics. Another limitation of this study is the ongoing lack of *in vivo* models to study the second phase of bacteremia, dissemination. The ability to measure dissemination separately from lung and splenic fitness would further define relevant interactions for each factor. Additionally, the present study investigates the hypervirulent KPPR1 strain yet classic *K*. *pneumoniae* strains may exhibit different or additional bacteremia fitness factors, especially given the large *Klebsiella* accessory genome [51]. For example, capsule type can predict interactions with liver-resident Kupffer cells. The KPPR1 K2 capsule may allow for *K*. *pneumoniae* evasion of uptake and killing by these cells, increasing tissue fitness in this site [52].

Bacteremia is a complex family of infections encompassing multiple sites and responses. Resident cells of the spleen vary widely from those in the liver, and nutrients limited at one site may be abundant in the other. Thus, it is necessary to define factors specifically enhancing bloodstream survival to better understand *K*. *pneumoniae* bacteremia. This study is the first to define *K*. *pneumoniae* splenic fitness factors in an *in vivo* mammalian system. Factors enhancing *K*. *pneumoniae* bacteremia are largely diverse yet represent functions conserved across primary sites and other Gram-negative species and may serve as attractive targets for future therapeutics.

## Materials and methods

### Ethics statement

This study was performed with careful adherence to humane animal handling guidelines [53] and approved by the University of Michigan Institutional Animal Care and Use Committee (protocol: PRO00009406).

### Murine Bacteremia

Mice used were between 6–12 weeks in age, and each experiment used male and female mice. Wild-type, *Ccr2*^*-/-*^ [45], and *Cybb*^*-/-*^ [46] mice from the C57BL/6 lineage were bred and maintained at the University of Michigan or directly purchased (Jackson Laboratory, Bar Harbor, ME). In each model of bacteremia, *K*. *pneumoniae* overnight cultures were centrifuged at 5,000xg for 15 minutes and pellets were resuspended in PBS. Cultures were adjusted to the correct concentration based on OD_600_ measurement. To model bacteremic pneumonia and intravascular bacteremia, mice were infected as previously described [16]. For pneumonia, animals were anesthetized with isoflurane and 50μL of PBS containing 1x10^6^ CFU of *K*. *pneumoniae* was retropharyngeally administered. For intravascular bacteremia, 100μL of PBS containing 1x10^5^ CFU of *K*. *pneumoniae* was administered by injection via the tail vein. Mice were sacrificed at 24 hours post-inoculation and lung, spleen, liver, or blood were collected. Cardiac punctures were used to obtain whole blood, which was dispensed into heparin coated tubes (BD, Franklin Lakes, NJ). After collection, organs were homogenized in PBS and bacterial burden was calculated by quantitative plating. When appropriate, competitive infections contained a 1:1 ratio of wild-type KPPR1 and a mutant of interest marked with antibiotic selection. Competitive indices were calculated by CFU using the following equation: (*mutant output/wild-type output)/(mutant input/wild-type input)*.

### Bacterial strains and reagents

Reagents were sourced from Sigma-Aldrich (St. Louis, MO) unless otherwise noted. *K*. *pneumoniae* strains were cultured overnight at 37°C with shaking in LB broth (Fisher Bioreagents, Ottowa, ON) or at 30°C on LB agar plates. Media were supplemented with 40μg/mL kanamycin to select for transposon mutants and isogenic knockout strains, or with 50μg/mL chloramphenicol to select for strains containing the plasmid pACYC184 and its derivatives. All bacterial strains in this study are detailed in S3 Table, and primers are detailed in S4 Table.

Complementation plasmids were generated as previously described [16]. The complementation vector, pACYC184 was linearized by BamHI and HindIII digestion (New England Biolabs, Ipswitch, MA). The locus for *arnD*, *purD*, *sspA*, *pdxA*, or *pdxA-ksgA-apaG-apaH*, along with upstream regions within 500 base pairs of the open reading frame predicted to contain the native promoter (predicted by SoftBerry BPROM; Softberry Inc, Mount Kisco, NY), were amplified from KPPR1 using primers with 5’ homology to linearized pACYC. For each gene, Gibson assembly was performed using the generated PCR products and linearized pACYC184 according to the manufacturer’s protocol with HiFi DNA Assembly Master Mix (New England Biolabs). The *pdxA*_operon_ amplicon and pACYC184 was ligated after digestion using T4 DNA ligase according to the manufacturer’s protocol (New England Biolabs). The Gibson or ligated product was transformed into *E*. *coli* TOP10 (New England Biolabs), and constructs were confirmed using full length plasmid sequencing (Plasmidsaurus, Eugene, OR). Verified plasmids were mobilized into *K*. *pneumoniae* by electroporation.

To generate a *pdxA* (VK055_2525) isogenic knockout, Lambda Red mutagenesis was performed as described [10,11,16,54]. Briefly, electrocompetent KPPR1 harboring the pKD46 plasmid was generated using an overnight culture grown at 30°C. The culture was diluted into LB broth with 50μg/mL spectinomycin, 50mM L-arabinose, 0.5mM EDTA (Promega, Madison, WI), and 10μM salicylic acid and grown at 30°C until exponential phase. Cells were cooled on ice for 30 minutes and then pelleted at 8,000xg for 15 minutes. Serial washes were performed at 4°C using 50mL 1mM HEPEs pH 7.4 (Gibco, Grand Island, NY), 50mL diH_2_O, and 20mL 10% glycerol. To generate site specific targets for *pdxA*, a kanamycin resistance cassette from the pKD4 plasmid was amplified with primers also containing 65 base pairs of homology at the 5’ end to the chromosome flanking the *pdxA* open reading frame (S4 Table). This fragment was electroporated into competent KPPR1-pKD46 and transformants were recovered overnight at 30°C, then selected on agar containing kanamycin after a 37° incubation. Knockouts were confirmed with colony PCR using primers flanking and internal to *pdxA*.

### Transposon insertion-site sequencing (TnSeq)

A previously described KPPR1 transposon library consisting of ~25,000 unique random insertions was used to generate four input pools [11,32]. To generate pools, the library was thawed, mixed, and 1mL was removed, pelleted, and resuspended in fresh PBS (Corning, Corning, NY). The OD_600_ was measured, and the library concentration adjusted to 4x10^3^ CFU/mL. 100uL of the adjusted library was plated to achieve a density of ~400 distinct colonies on individual plates. To achieve a desired complexity of 8,500 transposon mutants/pool (S2 Fig), 22 plates were scraped and the CFU combined into PBS. This was repeated four times to generate unique pools (Pools A-D), which were stored at -80°C until use.

Mice were inoculated with one of the four input pools at a dose of 1x10^6^ CFU/mouse in a volume of 100μL via tail vein injection [33]. After 24 hours, the mice were euthanized, and spleens were removed and homogenized in 2mL of PBS. Spleen colonization was determined based on quantitative culture using 100μL of homogenate. For each mouse the remaining homogenate was plated in 125μL increments on 100x15mm petri dishes (Corning), incubated at 37°C overnight, scraped, combined in ~125mL PBS, and mixed until homogenous. The OD_600_ was measured and 1x10^9^ CFU was removed, pelleted at 5,000xg for 15 minutes, supernatant removed, and the pellet stored at -80°C until DNA extraction. Any spleen with <8.5x10^3^ CFU/spleen was removed from the study as this colonization is lower than the inoculum mutant complexity and therefore may yield unreliable results. For each input and spleens with appropriate colonization, DNA was extracted from pellets using the Qiagen DNeasy UltraClean Microbial Kit (Qiagen, Hilden, DE) according to the manufacturer’s instructions. Purified DNA was submitted to the University of Minnesota Genomics Center for quality verification, library preparation, and sequencing [55]. Samples were sequenced using paired-end mode on a NovaSeq 6000 with a depth of 12 million reads/sample. Reads were mapped and normalized as previously described [56], and genes that influence bacteremia fitness were identified using the TnSeqDiff pipeline [57].

### Growth curves

To assess growth of *K*. *pneumoniae*, overnight cultures were adjusted to 1x10^7^ CFU/mL in the indicated medium. Using an Eon microplate reader and Gen5 software (Version 2.0, BioTek, Winooski, VT), OD_600_ was measured every 15 minutes and samples were incubated at 37°C with aeration for the duration of the experiment. Strains were measured in the following conditions: LB, M9 salts+20% active human serum, M9 salts+20% heat inactivated human serum, M9 salts+10% murine serum. Differences in growth were detected by area under the curve (AUC, GraphPad Prism Software, LaJolla, CA).

### Hypermucoviscosity

To measure hypermucoviscosity, 500μL of a 1.5mL LB overnight culture for each strain was added to 1.5mL fresh PBS. 900μL of the suspension was used to measure the OD_600_ (pre-spin) while the remaining suspension was centrifuged at 1,000xg for 5 minutes. The OD_600_ of the upper 900μL of supernatant was then measured (post-spin). Hypermucoviscosity = (post-spin)/(pre-spin).

### *Ex vivo* competition assays

Uninfected murine spleen and liver were homogenized in 2mL PBS and used for *ex vivo* competition assays as previously described [16]. Briefly, 90μL of homogenate and 10μL of PBS containing 1x10^4^ CFU of a 1:1 mixture of *K*. *pneumoniae* strains were combined and incubated at 37°C for three hours. CFU of each strain at t = 0 and t = 3 was measured by serial dilutions and quantitative culture from which competitive indices were generated.

### Oxidative stress survival assay

To define *K*. *pneumoniae* survival in the presence of oxidative stress, overnight bacterial cultures were adjusted to 1x10^7^ CFU/mL in PBS+1mM H_2_O_2_ and incubated for 2 hours at 37°C. Serial dilutions and quantitative culture defined the abundance of each strain before (t = 0) and after (t = 2) incubation. Percent survival was defined as [(CFU at t = 2)/(CFU at t = 0)]*100.

### Statistical analysis

All *in vivo* experiments were performed using at least two independent infections. All *in vitro* and *ex vivo* experiments were performed as independent biological replicates. Statistical significance was defined as a *p-*value <0.05 (GraphPad) as determined using: one-sample tests to assess differences from a hypothetical value of zero for competitive indices, unpaired and paired *t* tests to assess differences between two groups, or ANOVAs with Dunnett’s multiple comparisons to assess differences among multiple groups.

## Supporting information

S1 Fig Estimations of experimental bottlenecks in the tail-vein injection model of *K*. *pneumoniae* bacteremia. Estimation of *in vivo* bottlenecks were determined by competing KPPR1 against a neutral fitness transposon mutant (VK055_*1912*) at varying ratios in the tail vein injection model. The log_10_ competitive index at 24 hours post infection for individual mice is displayed with bars representing the median and interquartile range; **p<0.01 by one sample *t* test with a hypothetical value of 0. For each group, n≥9 mice in at least two independent infections.
(TIF)

S2 Fig KEGG orthology annotations for genes influencing *K*. *pneumoniae* bacteremia. (A) Primary KEGG annotations for the 74 genes defined as suppressing fitness. Secondary KEGG annotations for (B) the 58 genes increasing (from Fig 1), or (C) the 74 genes suppressing, *K*. *pneumoniae* bacteremia fitness. Number = total genes within each annotation, unclassified genes were not included in secondary annotation analysis.
(TIF)

S3 Fig Genes enhancing bacteremia are largely dispensable for *in vitro* replication and have differential effects on hypermucoviscosity. (A) *K*. *pneumoniae* strains with transposon mutations in genes influencing bacteremia were grown in LB and the OD_600_ was measured every 15 minutes for 12 hours. (B) Hypermucoviscosity was measured for each strain; hypermucoviscosity = (post-spin)/(pre-spin). In A-B, bars represent the mean value for each strain. *p<0.05, **p<0.01, ***p<0.001, ****p<0.0001 by one-way ANOVA with Dunnett’s multiple comparison for each strain compared to KPPR1; n = 5 in independent trials.
(TIF)

S4 Fig Insertion level analysis for distinct transposon mutations during bacteremia across five fitness factors. Read counts from TnSeq are displayed for unique transposon mutations contained within the genes (A) *arnD*, (B) *purD*, (C) *dsbA*, (D) *sspA*, or (E) *pdxA*. Closed circles indicate input read counts from one of four pools (Pools A-D); open circles indicate read counts from recovered from the spleen 24 hours post infection for individual mice.
(TIF)

S5 Fig CFU summary for tail vein injections. Five factors indicated by TnSeq as significantly enhancing bacteremia were selected for *in vivo* validation using the tail vein injection model. The 1:1 inoculum consisted of KPPR1 and transposon mutants for (A) *arnD*, (B) *purD*, (C) *dsbA*, (D) *sspA*, or (E) *pdxA*. Competitions were also performed using strains carrying the empty pACYC vector (_ev_) within KPPR1 and (F) *arnD*, (H) *dsbA*, or (J) *sspA*. Complementation was provided on pACYC under control of the native promoter of (G) *arnD* (*arnD*+pACYC_*arnD*_), (I) *dsbA* (*dsbA*+pACYC_*dsbA*_), or (K) *sspA* (*sspA*+pACYC_*sspA*_). The log_10_ CFU burden in the spleen and liver at 24 hours post infection is displayed, corresponding to competitive indices in Fig 2. *p<0.05, **p<0.01, ***p<0.001 by paired *t* test with Holm-Sidak multiple comparison. For each group, n*≥*8 mice in at least two independent infections.
(TIF)

S6 Fig Summary for additional statistics. A fitness neutral KPPR1 strain marked with a kanamycin resistance cassette was competed against wild-type KPPR1 in the (A-B) tail vein injection model, (C-E) within *ex vivo* organ homogenate, or (F-H) in the bacteremic pneumonia model. Unpaired *t* tests compared the competitive index for the fitness neutral competition, KPPR1:KPPR1_Kan_, against the experimental results from (A-B) Fig 2, (C-E) Fig 4, or (F-H) Fig 5. For all, *p<0.05, **p<0.01, ***p<0.001, ****p<0.0001. For all, the log_10_ competitive index at 24 hours post infection is displayed for individual mice with bars representing the median and interquartile range.
(TIF)

S7 Fig Area under the curve values for *K*. *pneumoniae* growth in serum. Area under the curve (AUC) was calculated for the growth of individual strains in each condition represented in Fig 3. *K*. *pneumoniae* strains were grown in M9 salts supplemented with (A) 10% mouse serum or 20% human serum that was either (B) active or (C) heat inactivated. *K*. *pneumoniae* strains carrying the empty vector pACYC (_ev_) or pACYC expressing (D,E) *purD* (*purD*+pACYC_*purD*_) or (F) *pdxA* (Δ*pdxA*+pACYC_*pdxA*_) were grown in 10% mouse serum (D, F). Chemical complementation for *purD* was measured by supplementation of 1mM purines prior to growth (E). For all, the OD_600_ was measured every 15 minutes for 12 hours. Differences in growth compared to KPPR1 or KPPR1_ev_ were detected by area under the curve using a one-way ANOVA with Dunnett’s multiple comparison for each strain compared to wild-type; *p<0.05, **p<0.01, ***p<0.001, ****p<0.0001. For each, n = 3 in independent trials and bars represent the mean AUC for each group.
(TIF)

S8 Fig CFU summary for *ex vivo* competitions. *K*. *pneumoniae* strains were competed at a 1:1 ratio in organ homogenate generated from uninfected mice. An input of 1x10^4^ CFU was added to each well and incubated at 37°C for 3 hours. Log_10_ CFU/well for each strain at the start (t = 0) and end (t = 3) of the incubation are displayed, corresponding to competitive indices in Fig 4. n≥3 competitions in mouse organs from single animals in independent trials, and bars represent the mean log_10_ CFU for each organ.
(TIF)

S9 Fig CFU summary for bacteremic pneumonia. To model bacteremic pneumonia, mice were infected with 1x10^6^ CFU *K*. *pneumoniae*. Competitive infections were performed with a 1:1 mixture of KPPR1 and transposon mutants for (A) *arnD*, (B) *purD*, (C) *dsbA*, (D) *sspA*, or (E-H) *pdxA* or (I-L) a *pdxA* knockout (Δ*pdxA*). Competitions were performed with strains carrying the pACYC vector (_ev_) or *pdxA* complementation provided on pACYC under control of the native promoter for *pdxA* only (F, H, K; pACYC_*pdxA*_) or *pdxA* and downstream members of the operon (L; pACYC_operon_). The log_10_ bacterial burden at 24 hours post infection is displayed corresponding to competitive indices in Fig 5. For (A-E, G-L), *p<0.05, **p<0.01, ***p<0.001, ****p<0.0001 by paired *t* test with Holm-Sidak multiple comparison. For (F) no comparisons were significant between competitive indices within each tissue by unpaired *t* test, and the log_10_ competitive index at 24 hours post infection is displayed for individual mice with bars representing the median and interquartile range. For each group, n≥10 mice in at least two independent infections.
(TIF)

S10 Fig CFU summary for *in vitro* and *in vivo* oxidative stress resistance. (A) Resistance to oxidative stress was measured by incubating *K*. *pneumoniae* strains with H_2_O_2_. Complementation was performed by comparing strains carrying empty pACYC (_ev_) to those with pACYC expression of (B) *sspA* (*sspA*+pACYC_*sspA*_) or (C) *pdxA* (*pdxA*+pACYC_*pdxA*_). For (A-C), log_10_ CFU/mL is displayed. (D-H) In a model of bacteremic pneumonia, mice were infected with 1x10^6^ CFU *K*. *pneumoniae* containing a 1:1 mix of KPPR1 and a transposon mutant for (D) *dsbA*, (E) *sspA*, or (F) *pdxA* in *Ccr2*^*-/-*^ mice and (G) *sspA* or (H) Δ*pdxA* in *Cybb*^-*/-*^ mice. For (A-F), *p<0.05, **p<0.01, ***p<0.001, ****p<0.0001 by paired *t* test with Holm-Sidak multiple comparison. For (G,H), **p<0.01, ***p<0.001, ****p<0.0001 by unpaired *t* test. For each group, n≥7 mice in two independent infections. In A-C and G-H, bars represent the mean log_10_ CFU.
(TIF)

S1 Table TnSeq results. Results from the TnSeq screen for each gene represented in the study.
(XLSX)

S2 Table Genes in which mutations increase fitness. Genes in which transposon mutations resulted in higher recovery after bacteremia.
(XLSX)

S3 Table Strain list. Bacterial strains used in this study.
(XLSX)

S4 Table Primer list. Primers used in this study.
(XLSX)
