# Supplementary figures and images for: Klebsiella pneumoniae causes bacteremia using factors that mediate tissue-specific fitness and resistance to oxidative stress
Source: PLoS Pathog. 2023 Jul 18;19(7):e1011233. doi: 10.1371/journal.ppat.1011233 (PMC10381055; doi:10.1371/journal.ppat.1011233)

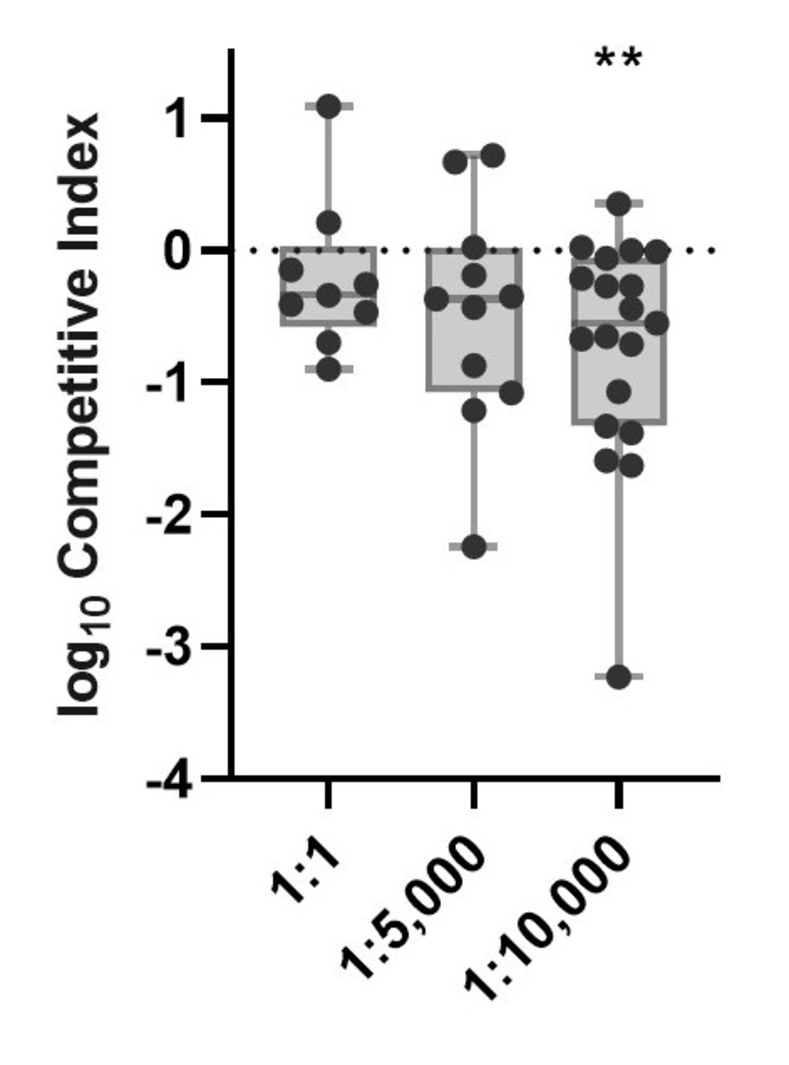

Supplement: S1 Fig — Estimation of in vivo bottlenecks were determined by competing KPPR1 against a neutral fitness transposon mutant (VK055_1912) at varying ratios in the tail vein injection model. The log10 competitive index at 24 hours post infection for individual mice is displayed with bars representing the median and interquartile range; **p<0.01 by one sample t test with a hypothetical value of 0. For each group, n≥9 mice in at least two independent infections. (TIF) [file ppat.1011233.s001.tif]

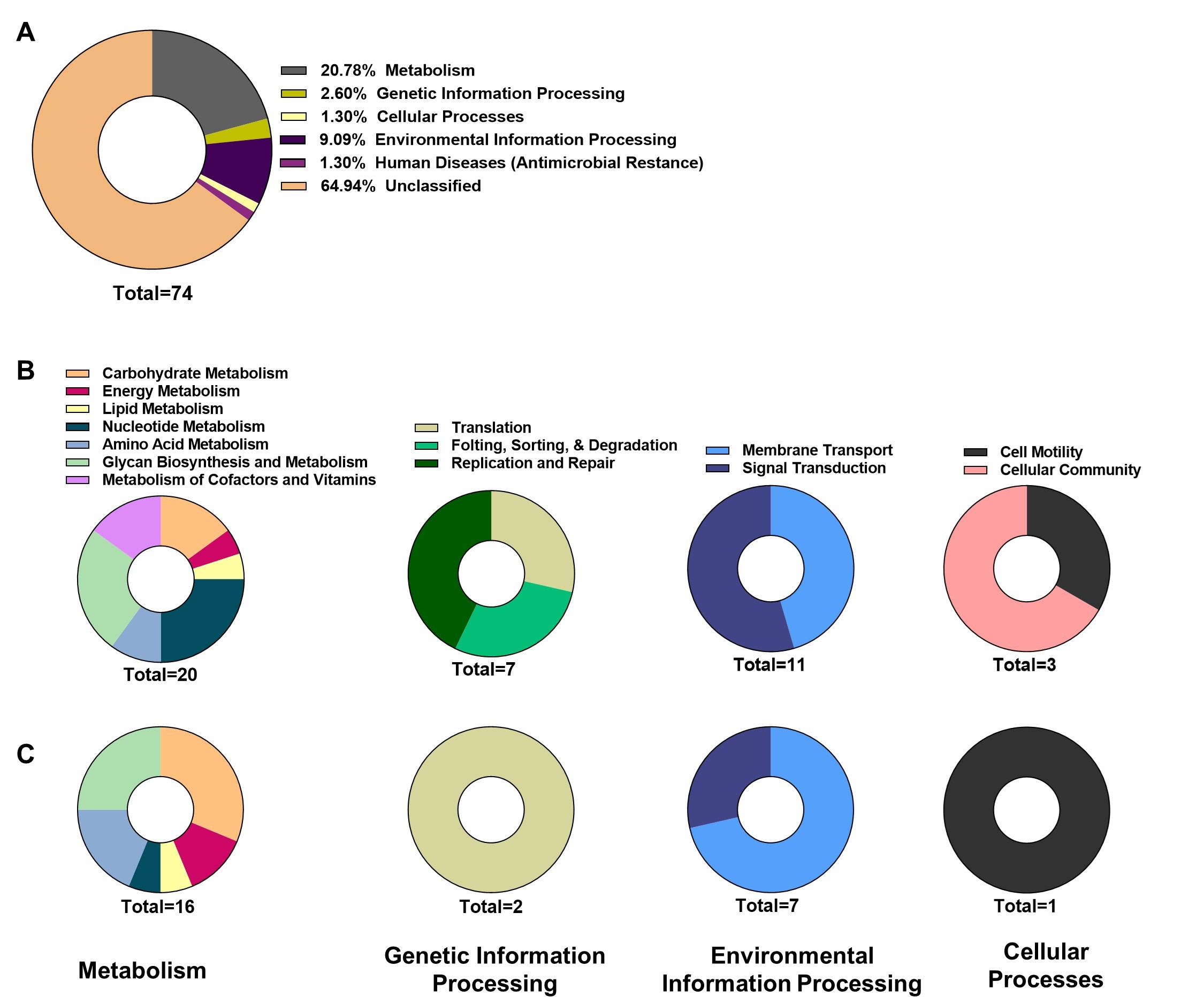

Supplement: S2 Fig — (A) Primary KEGG annotations for the 74 genes defined as suppressing fitness. Secondary KEGG annotations for (B) the 58 genes increasing (from Fig 1), or (C) the 74 genes suppressing, K. pneumoniae bacteremia fitness. Number = total genes within each annotation, unclassified genes were not included in secondary annotation analysis. (TIF) [file ppat.1011233.s002.tif]

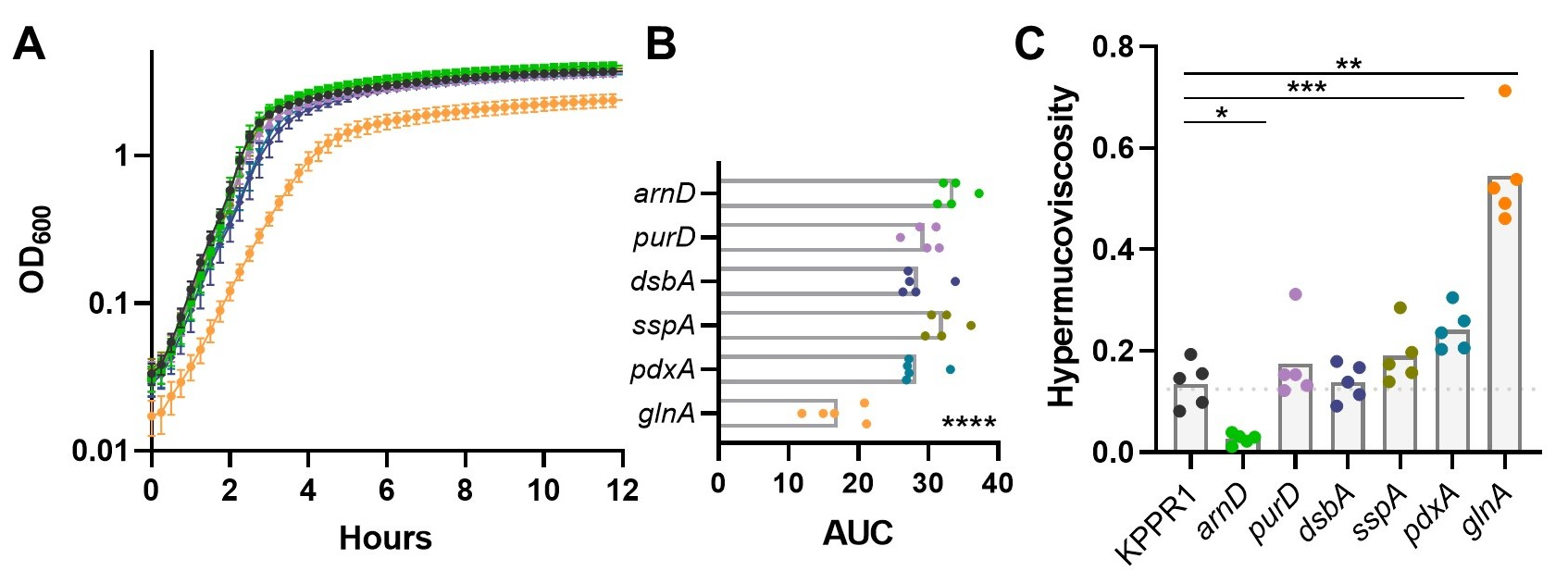

Supplement: S3 Fig — (A) K. pneumoniae strains with transposon mutations in genes influencing bacteremia were grown in LB and the OD600 was measured every 15 minutes for 12 hours. (B) Hypermucoviscosity was measured for each strain; hypermucoviscosity = (post-spin)/(pre-spin). In A-B, bars represent the mean value for each strain. *p<0.05, **p<0.01, ***p<0.001, ****p<0.0001 by one-way ANOVA with Dunnett’s multiple comparison for each strain compared to KPPR1; n = 5 in independent trials. (TIF) [file ppat.1011233.s003.tif]

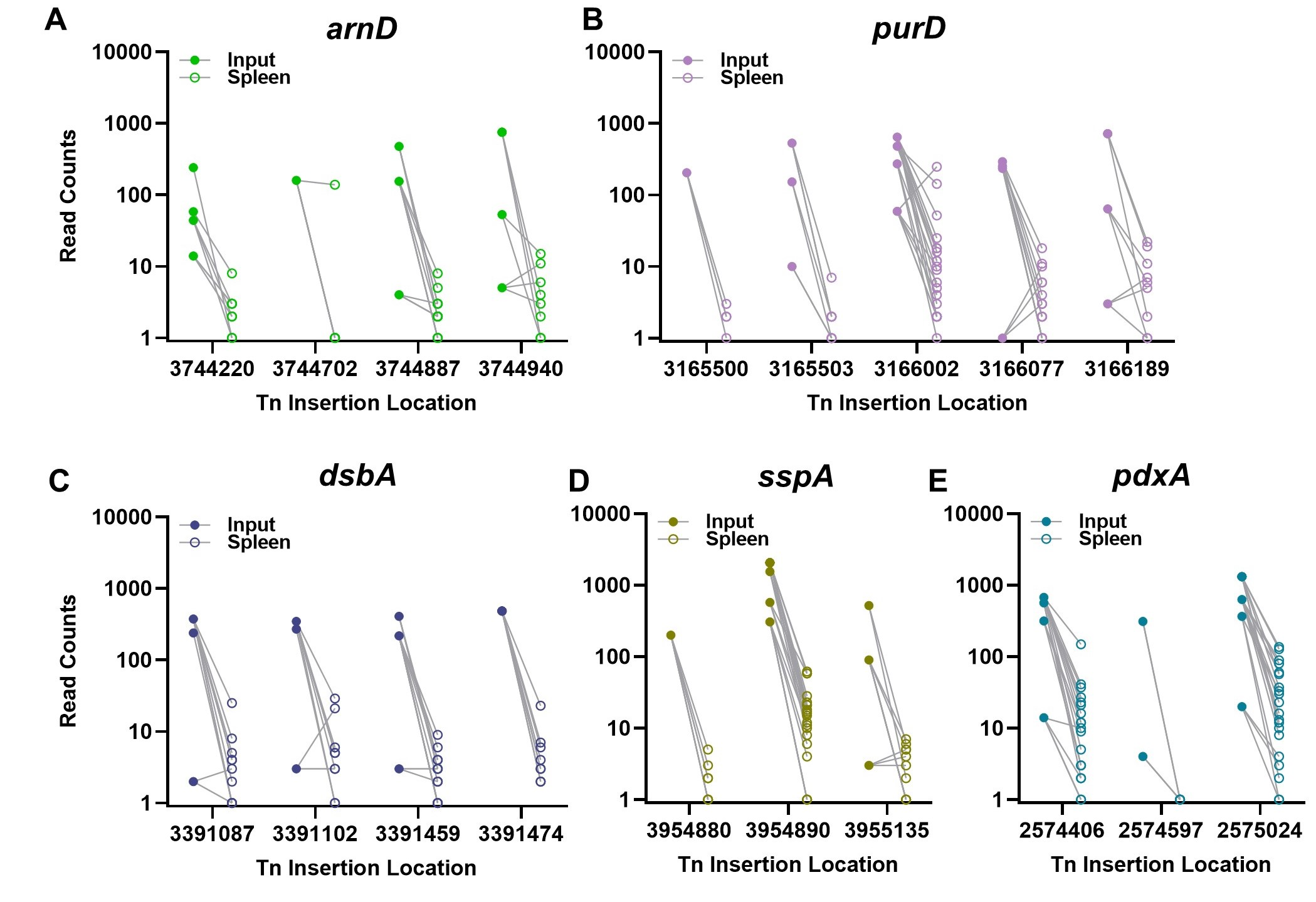

Supplement: S4 Fig — Read counts from TnSeq are displayed for unique transposon mutations contained within the genes (A) arnD, (B) purD, (C) dsbA, (D) sspA, or (E) pdxA. Closed circles indicate input read counts from one of four pools (Pools A-D); open circles indicate read counts from recovered from the spleen 24 hours post infection for individual mice. (TIF) [file ppat.1011233.s004.tif]

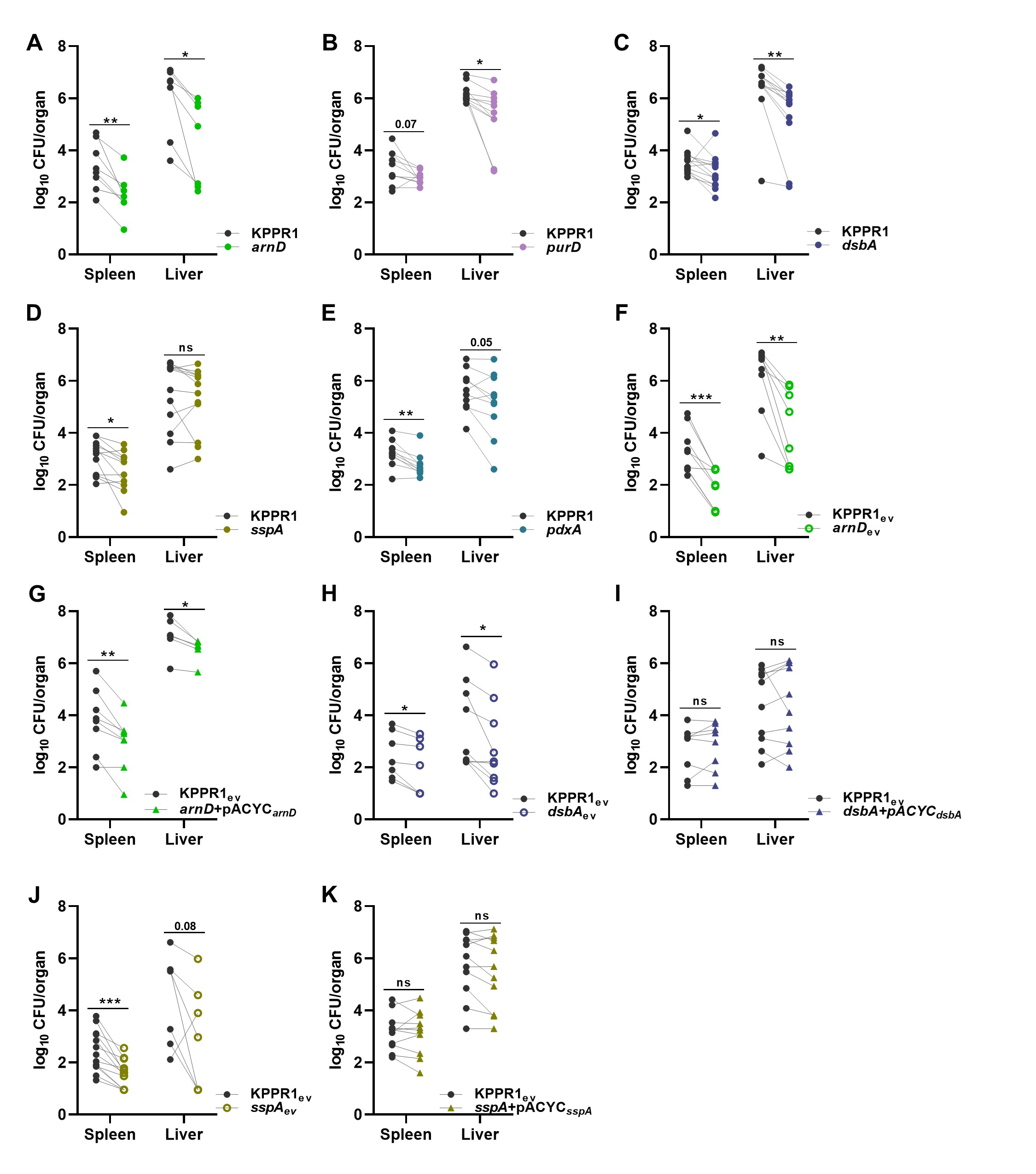

Supplement: S5 Fig — Five factors indicated by TnSeq as significantly enhancing bacteremia were selected for in vivo validation using the tail vein injection model. The 1:1 inoculum consisted of KPPR1 and transposon mutants for (A) arnD, (B) purD, (C) dsbA, (D) sspA, or (E) pdxA. Competitions were also performed using strains carrying the empty pACYC vector (ev) within KPPR1 and (F) arnD, (H) dsbA, or (J) sspA. Complementation was provided on pACYC under control of the native promoter of (G) arnD (arnD+pACYCarnD), (I) dsbA (dsbA+pACYCdsbA), or (K) sspA (sspA+pACYCsspA). The log10 CFU burden in the spleen and liver at 24 hours post infection is displayed, corresponding to competitive indices in Fig 2. *p<0.05, **p<0.01, ***p<0.001 by paired t test with Holm-Sidak multiple comparison. For each group, n≥8 mice in at least two independent infections. (TIF) [file ppat.1011233.s005.tif]

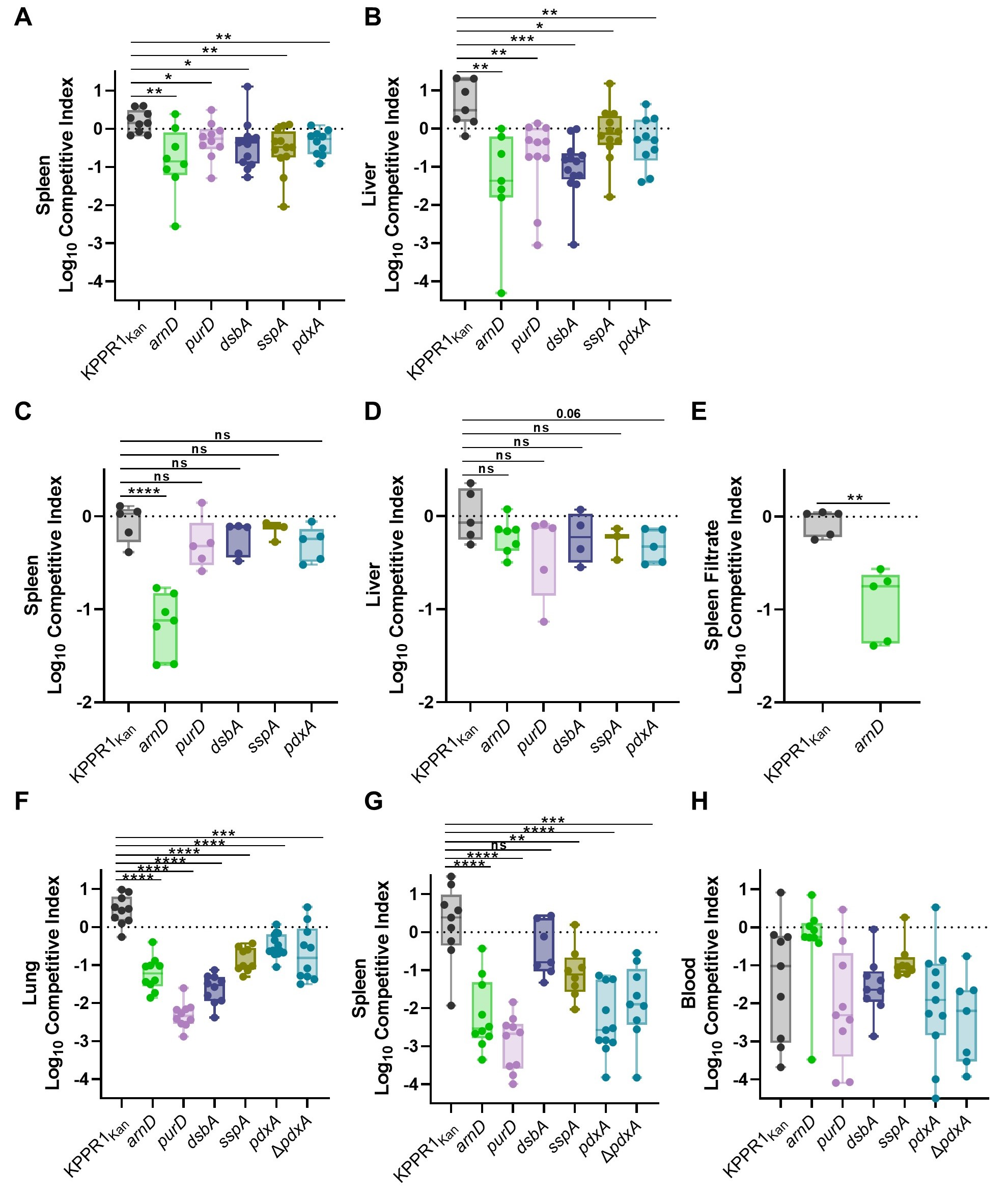

Supplement: S6 Fig — A fitness neutral KPPR1 strain marked with a kanamycin resistance cassette was competed against wild-type KPPR1 in the (A-B) tail vein injection model, (C-E) within ex vivo organ homogenate, or (F-H) in the bacteremic pneumonia model. Unpaired t tests compared the competitive index for the fitness neutral competition, KPPR1:KPPR1Kan, against the experimental results from (A-B) Fig 2, (C-E) Fig 4, or (F-H) Fig 5. For all, *p<0.05, **p<0.01, ***p<0.001, ****p<0.0001. For all, the log10 competitive index at 24 hours post infection is displayed for individual mice with bars representing the median and interquartile range. (TIF) [file ppat.1011233.s006.tif]

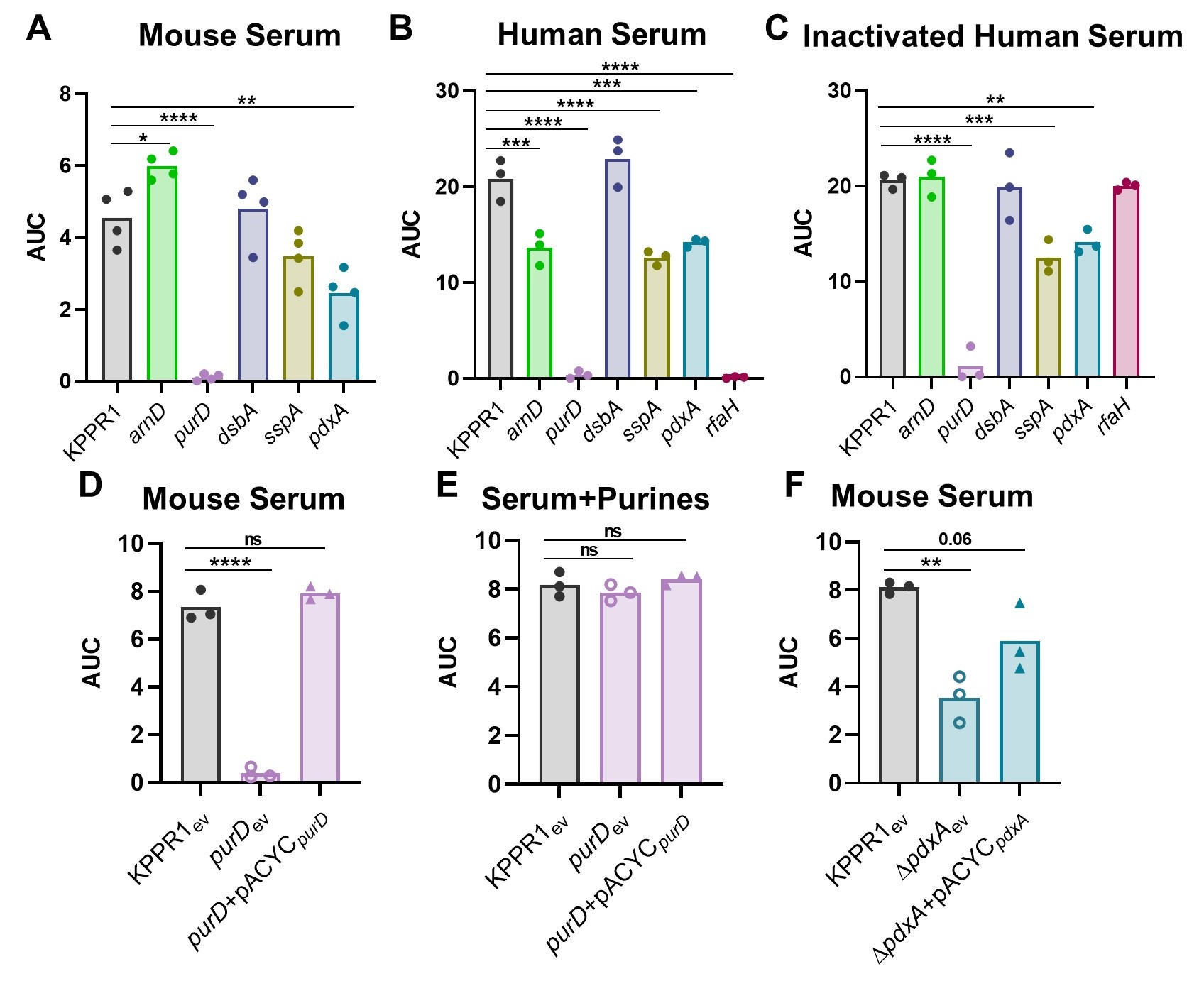

Supplement: S7 Fig — Area under the curve (AUC) was calculated for the growth of individual strains in each condition represented in Fig 3. K. pneumoniae strains were grown in M9 salts supplemented with (A) 10% mouse serum or 20% human serum that was either (B) active or (C) heat inactivated. K. pneumoniae strains carrying the empty vector pACYC (ev) or pACYC expressing (D,E) purD (purD+pACYCpurD) or (F) pdxA (ΔpdxA+pACYCpdxA) were grown in 10% mouse serum (D, F). Chemical complementation for purD was measured by supplementation of 1mM purines prior to growth (E). For all, the OD600 was measured every 15 minutes for 12 hours. Differences in growth compared to KPPR1 or KPPR1ev were detected by area under the curve using a one-way ANOVA with Dunnett’s multiple comparison for each strain compared to wild-type; *p<0.05, **p<0.01, ***p<0.001, ****p<0.0001. For each, n = 3 in independent trials and bars represent the mean AUC for each group. (TIF) [file ppat.1011233.s007.tif]

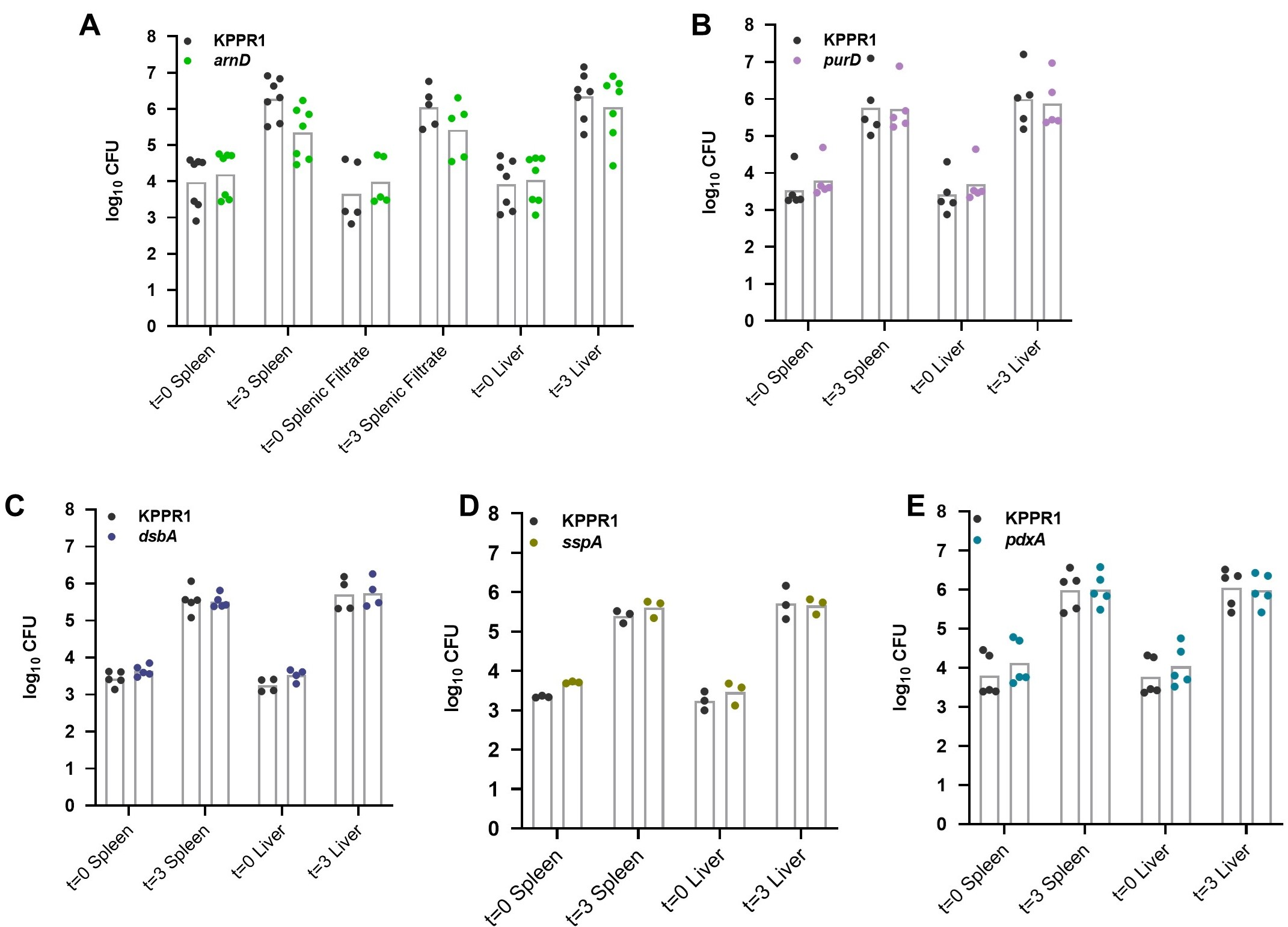

Supplement: S8 Fig — K. pneumoniae strains were competed at a 1:1 ratio in organ homogenate generated from uninfected mice. An input of 1x10^4^ CFU was added to each well and incubated at 37°C for 3 hours. Log10 CFU/well for each strain at the start (t = 0) and end (t = 3) of the incubation are displayed, corresponding to competitive indices in Fig 4. n≥3 competitions in mouse organs from single animals in independent trials, and bars represent the mean log10 CFU for each organ. (TIF) [file ppat.1011233.s008.tif]

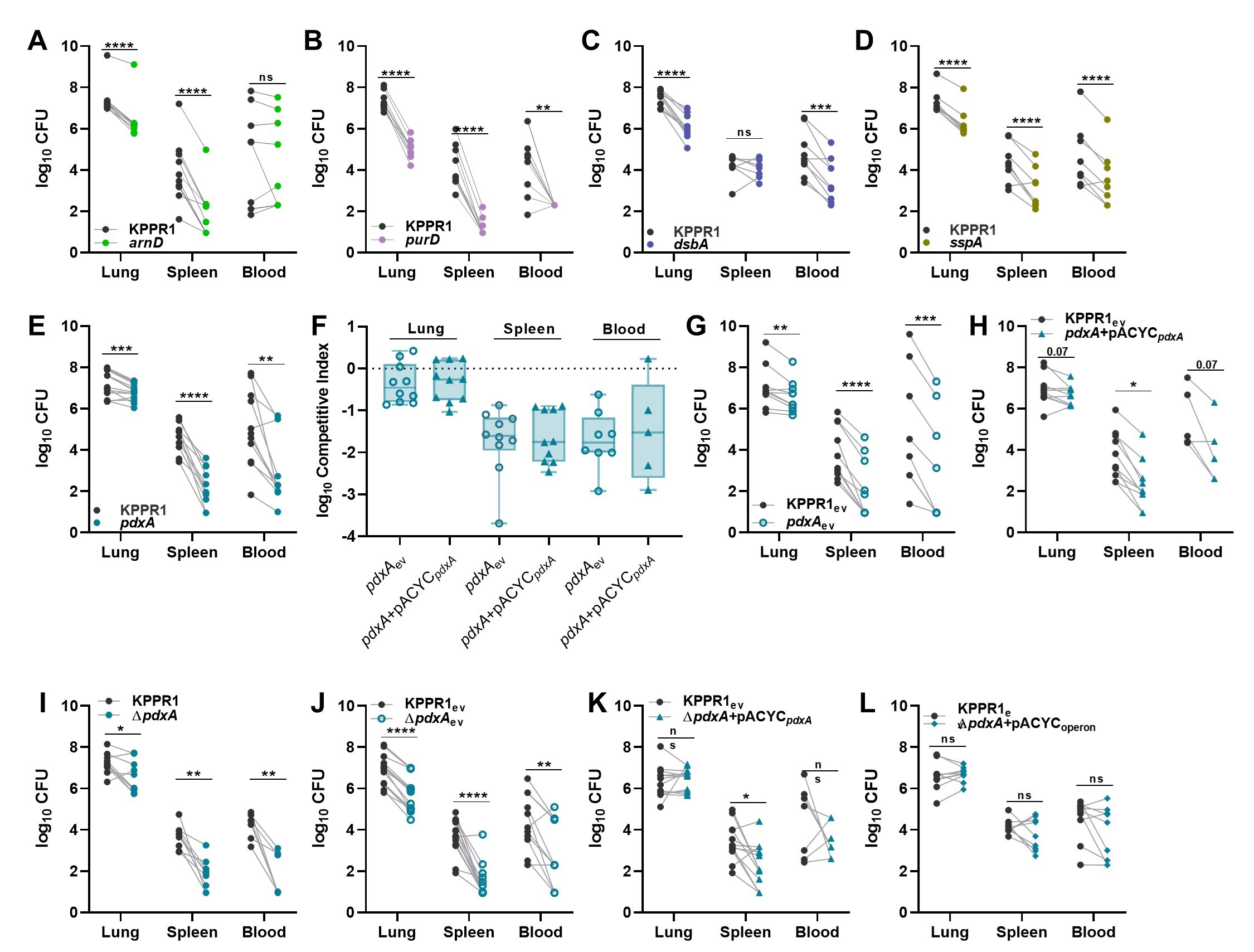

Supplement: S9 Fig — To model bacteremic pneumonia, mice were infected with 1x10^6^ CFU K. pneumoniae. Competitive infections were performed with a 1:1 mixture of KPPR1 and transposon mutants for (A) arnD, (B) purD, (C) dsbA, (D) sspA, or (E-H) pdxA or (I-L) a pdxA knockout (ΔpdxA). Competitions were performed with strains carrying the pACYC vector (ev) or pdxA complementation provided on pACYC under control of the native promoter for pdxA only (F, H, K; pACYCpdxA) or pdxA and downstream members of the operon (L; pACYCoperon). The log10 bacterial burden at 24 hours post infection is displayed corresponding to competitive indices in Fig 5. For (A-E, G-L), *p<0.05, **p<0.01, ***p<0.001, ****p<0.0001 by paired t test with Holm-Sidak multiple comparison. For (F) no comparisons were significant between competitive indices within each tissue by unpaired t test, and the log10 competitive index at 24 hours post infection is displayed for individual mice with bars representing the median and interquartile range. For each group, n≥10 mice in at least two independent infections. (TIF) [file ppat.1011233.s009.tif]

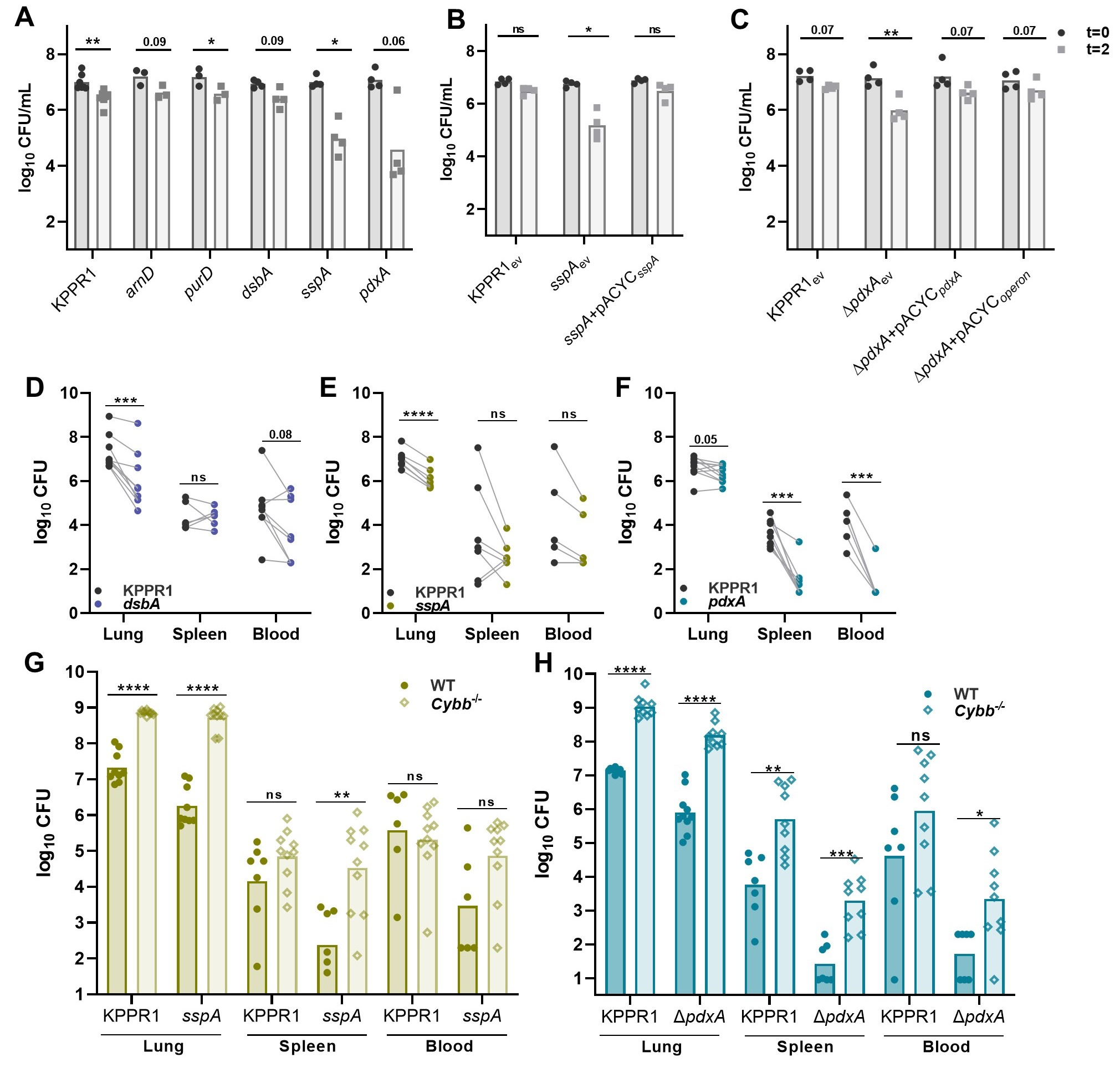

Supplement: S10 Fig — (A) Resistance to oxidative stress was measured by incubating K. pneumoniae strains with H2O2. Complementation was performed by comparing strains carrying empty pACYC (ev) to those with pACYC expression of (B) sspA (sspA+pACYCsspA) or (C) pdxA (pdxA+pACYCpdxA). For (A-C), log10 CFU/mL is displayed. (D-H) In a model of bacteremic pneumonia, mice were infected with 1x10^6^ CFU K. pneumoniae containing a 1:1 mix of KPPR1 and a transposon mutant for (D) dsbA, (E) sspA, or (F) pdxA in Ccr2-/- mice and (G) sspA or (H) ΔpdxA in Cybb-/- mice. For (A-F), *p<0.05, **p<0.01, ***p<0.001, ****p<0.0001 by paired t test with Holm-Sidak multiple comparison. For (G,H), **p<0.01, ***p<0.001, ****p<0.0001 by unpaired t test. For each group, n≥7 mice in two independent infections. In A-C and G-H, bars represent the mean log10 CFU. (TIF) [file ppat.1011233.s010.tif]
